# Frugal Sampling Strategies for Navigating Complex Reaction Spaces

**DOI:** 10.1021/acs.oprd.6c00027

**Published:** 2026-04-10

**Authors:** Vincent Porte, Luca Hepp, Philipp Kollmus, Shizhao Lu, Eloisa Serrano, Daniela Blanco, Marco Santagostino

**Affiliations:** † Chemical Development Germany, Boehringer Ingelheim Pharma GmbH & Co. KG, Biberach an der Riß 88397, Germany; ‡ Sunthetics, Inc, 3055 Hunter Rd, San Marcos, Texas 78666, United States

**Keywords:** HTE, frugal sampling, cross-coupling, DoE, Bayesian optimization

## Abstract

As the complexity of the chemical space continues to grow, innovative strategies are required to enhance screening efficiency. With the main goal of identifying overarching trends, we introduce novel high-throughput experimentation (HTE) sampling strategies that explore broader chemical spaces and only require 25% of the experiments compared to full factorial designs. Our carefully designed quasi-random sampling methods maximize plate layout diversity while preserving practicality for 96-well microtiter plates. The frugal samplings were successfully applied to four challenging metal-catalyzed cross-coupling reactions. The resulting screens retained sufficient information to understand the main tendencies and enable informed decision-making for subsequent optimization activities. In one case study, the method was coupled with a Bayesian optimization workflow to enable cost-effective transformations within the explored chemical space by introducing a cost-penalized yield as an optimization objective.

## Graphical abstract

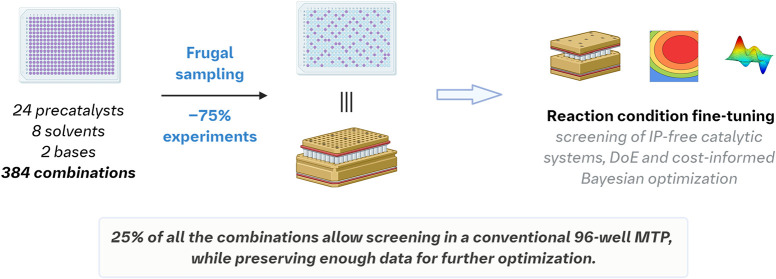

## Introduction

The steady increase in the complexity of target molecules in medicinal chemistry programs calls for innovative approaches to optimize synthetic procedures quickly and consistently.^1^ Owing to its ability to maximize experimental efficiency, high-throughput experimentation (HTE) has proven instrumental in extracting precious information and has become one of the preferred tools in many companies, as well as in some academic groups, when approaching multidimensional optimization problems.^2–7^ Historically, for a given transformation, HTE has relied on grid-based plating schemes, where the combinations of reagents (catalysts, additives, solvents...) are systematically tested in a full factorial design to derive meaningful trends. The grid-sampling strategy remains broadly implemented due to the practical compatibility with multichannel instruments or electronic pipettes for dispensing reagent stock solutions and solvents into 96-well microtiter plates (MTPs).

Building on the grid-based approach, we, at Boehringer Ingelheim, and others have established primary screenings consisting of a standardized set of carefully curated conditions highly likely to provide starting points for optimization (Figure 1A).^8–11^ These screenings are designed to cover key parameters for the most commonly encountered reactions in the pharmaceutical industry, including both metal- and nonmetal-catalyzed transformations.^12^ While primary screening plates are designed to identify the most relevant categorical parameters and assess reactivity trends, they are typically followed by a more detailed investigation of the most promising (catalytic) systems, here referred to as secondary screenings. This may involve an in-depth assessment of solvent, base or additive effects. The final stages of the optimization process can include Design of Experiments (DoE) investigations, complemented by preliminary kinetic studies and early assessments of process stability against adventitious oxygen and water traces. The overarching goal of this workflow is to deliver safe, sustainable, and cost-effective processes built around IP-free (catalytic) components, which are directly applicable at preparative scale for preclinical and early clinical drug substance deliveries with minimal need for further optimization.

**Figure 1:**
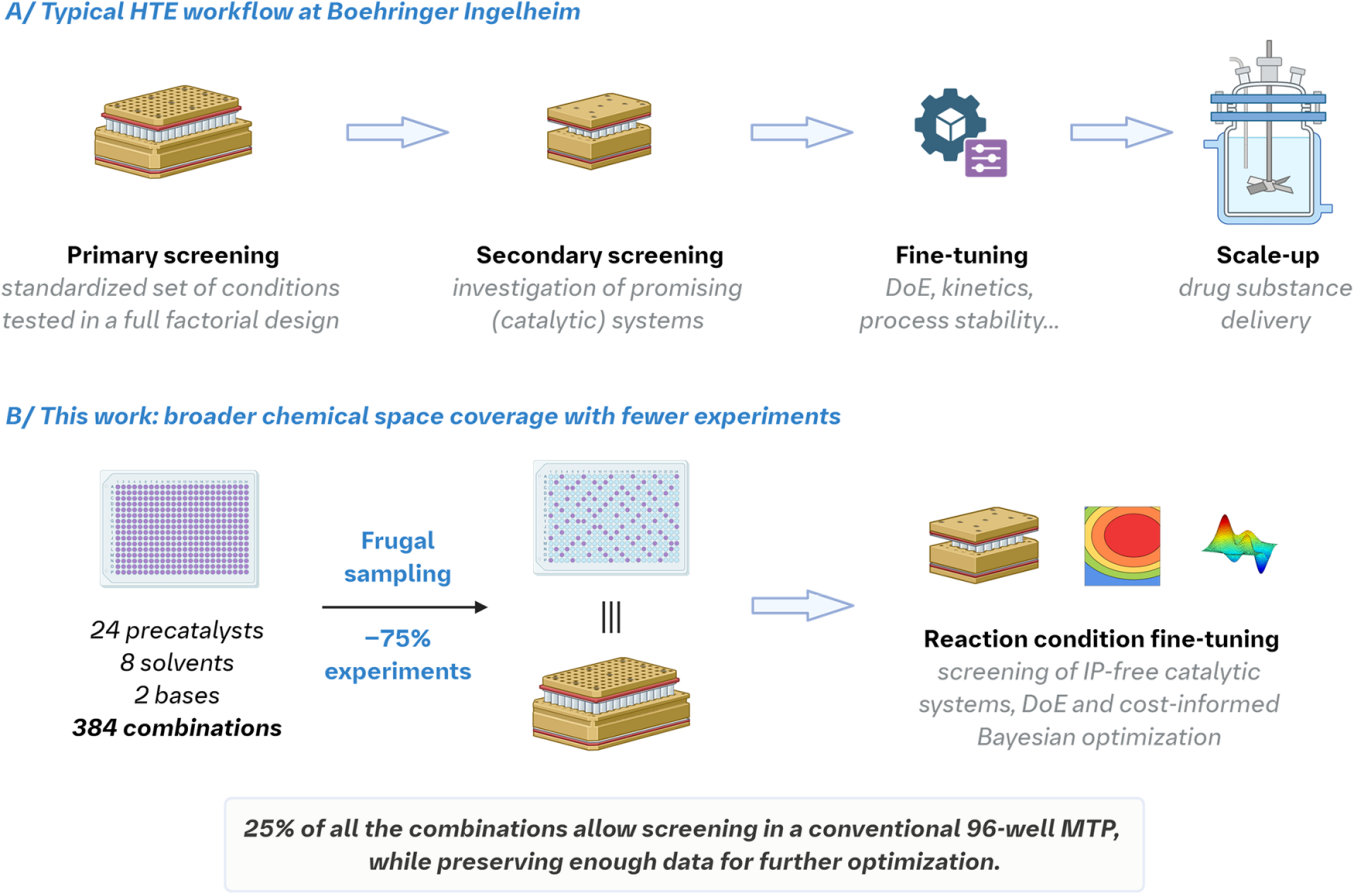
(A) Typical HTE workflow at Boehringer Ingelheim. (B) The improved strategy to optimize complex chemical transformations. The figure was created in Biorender.^13^

As the chemical space becomes increasingly complex, we and others have developed complementary strategies to improve screening efficiency. One solution has been to increase the number of reactions conducted per HTE campaign by employing 384- or 1536-well MTPs.^14–18^ However, these formats often lack the flexibility and operational practicality of the widely adopted 96-well MTPs. An opposite strategy has consisted in screening mixtures of plausible candidates, usually carried out in a 24-well format, followed by deconvolution steps to identify the active species (pool-and-split).^19–22^ While pool-and-split protocols are routinely used in our screening laboratories, they have poor initial resolution, and promising conditions may be overlooked. Beyond those strategies, considerable efforts have been dedicated to predicting improved reaction conditions and designing smarter screening strategies, driven by data science and advanced computational techniques.^23–26^ Although machine learning can offer valuable insights, it typically requires substantial experimental efforts to generate an initial data set and often suffers from limited generalizability.

Reflecting on over a decade of HTE experimentation in our facility, we have observed that many primary screenings can be analyzed by examining overall trends, rather than focusing on individual reaction outcomes. This is not entirely surprising, given the intrinsic redundancy contained in the full factorial HTE plate designs. For metal-catalyzed primary screenings, identical solvent/base combinations are often tested across multiple ligands, or the same precatalyst may be evaluated several times within a single plate. While this redundancy supports more reliable decision-making in early stage optimization, the classical grid-based design faces significant limitations when applied to very large design spaces. We hypothesized that a carefully selected subset of categorical factor combinations (96 or multiple thereof) could yield sufficient insights to guide subsequent optimization efforts.

Here, we present novel strategies to probe broad chemical spaces (384 or multiple thereof) using a limited number of experiments, with the main goal of identifying reactivity trends during the initial screening phase (Figure 1B). By employing frugal, quasi-random sampling techniques reminiscent of a fractional factorial design in DoE, the 96-well MTP layouts enable coverage of a significantly wider chemical space while reducing the number of experiments by 75% compared to a full factorial design. Compared to other space-filling sampling strategies, such as Sobol and Latin hypercube, the frugal samplings presented here were engineered to maximize diversity and ensure a balanced distribution of the categorical parameters, while preserving the experimental practicality of grid-like plating approaches in 96-well formats. We disclose the layouts of explorative plates for addressing Suzuki–Miyaura and Buchwald-Hartwig transformations and demonstrate their application across four case studies. The screens retain sufficient information to guide further optimization activities, which are carried out to fulfill the goals of chemical process development. In one example, we illustrate how the reduced sampling approach can be coupled with a Bayesian optimization (BO) workflow to meet not only reaction performance but also lower the cost of the goods, enabling cost-effective transformations within the explored chemical space.

## Results and Discussion

### Frugal Sampling Strategy

In recent years, the rapid growth of available ligands has rendered their selection increasingly challenging, especially in combination with the evaluation of other factors (e.g., solvent, bases).^27–30^ For Pd-catalyzed primary screenings, promising ligands can be identified using readily activated ligand-Pd precomplexes (**PXXX_PdXX**). This approach circumvents the need for in situ generation of active species, which can be inconsistent and strongly dependent on the ligand and the Pd source employed.^31–33^ For example, P­(*t*Bu)_3_Pd­(crotyl)Cl is used instead of combining P­(*t*Bu)_3_ with a Pd source (e.g., Pd­(OAc)_2_ or Pd_2_(dba)_3_).^34^ Despite the advantages offered by ligand-Pd precomplexes, a screening involving 24 precatalysts, 8 solvents and 2 bases remains resource-intensive, requiring the evaluation of 384 combinations. With the main objective of identifying overarching trends within a 96-well format, we envisioned a minimalist sampling approach designed to reduce redundancy, while maintaining a grid-like layout wherever possible to facilitate experimental setup. To implement this frugal sampling method, the eight solvents and two bases were initially arranged according to a conventional 96-well layout (as if the design included only 6 precatalysts) to ensure their practical plating (Figure 2). Then, to achieve a balanced distribution, each of the 24 precatalysts (abbreviated as **PXXX** in Figure 2) must be employed four times (96 wells/24 precatalysts = 4); paired twice with each base and cannot be used more than once in each solvent, resulting in four unique base-solvent pairs. A custom script was developed to assign the precatalysts to the wells to fulfill these constraints (see Supporting Information, section 2 and additional spreadsheet). Specifically, the 96-well MTP was partitioned into four quadrants of 24 wells. Then, the 24 precatalysts were distributed into each quadrant using a combination of randomized allocation and brute-force methods (guess, test, repeat). From a practical standpoint, we anticipated that solvents and bases (with inorganic bases as aqueous solution) could be readily dispensed using electronic pipettes. For nonarray-based precatalyst plating, automated powder dispensing platforms should be considered.^35^ In our case, we leveraged the ChemBeads technology along with the rapid dispensing capabilities of our recently reported solid dosing robot to ensure accurate and traceable dosing.^16,36^

**Figure 2:**
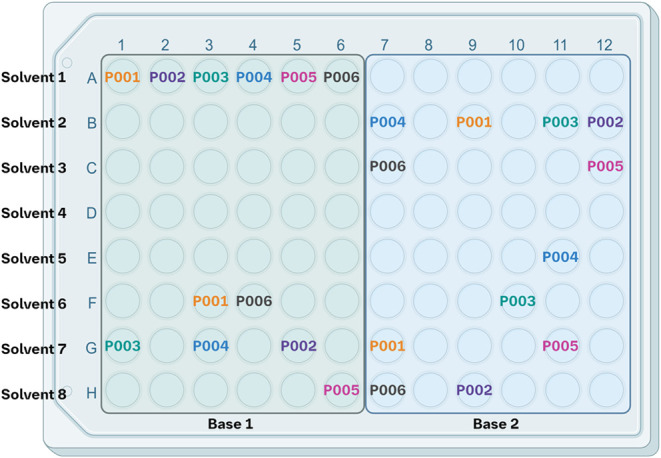
Frugal sampling plating scheme for a primary screening employing 24 precatalysts (abbreviated as **PXXX**; illustrated here only for 6 examples for clarity), 8 solvents and 2 bases. Each precatalyst is exposed twice to each base and no precatalyst appears more than once in a solvent row. The figure was created in Biorender.^13^

### C­(sp^2^)-C­(sp^2^) Suzuki-Miyaura Coupling

To validate our assumption that a limited subset of all possible combinations of categorical factors could provide sufficient insights for further optimization, we selected four challenging cross coupling reactions. In the first example, we examined the Suzuki-Miyaura coupling between 5-bromoindole (**1**) and 2,6-difluorophenyl boronic acid (**2**) at ambient temperature (Figure 3). The latter is an aryl boronic acid prone to protodeboronation under basic conditions.^37–40^ Compared to our standard conditions for this type of coupling, we expanded our historical ligand set from 12 to 24 candidates (see Supporting Information for details; section 3a), incorporating recent developments from the literature. While our primary screening typically relies on an aqueous solution of K_2_CO_3_, we introduced a weaker organic base, such as *N*,*N*-diisopropylethylamine (DIPEA), to potentially modulate reactivity and mitigate protodeboronation.^40,41^ For solvent selection, we maintained our usual 8 solvents, covering a broad range of polarities. To study this complex chemical space within a 96-well MTP (24 × 8 × 2 = 384 combinations), we applied the frugal sampling described previously and illustrated in Figure 2. Based on the screening results, we observed that DIPEA consistently outperformed K_2_CO_3_, particularly in combination with acetonitrile (ACN), where four out of six precatalysts (e.g., **P009_Pd5 | QPhos**) yielded high conversions and yields (Figure 3).

**Figure 3:**
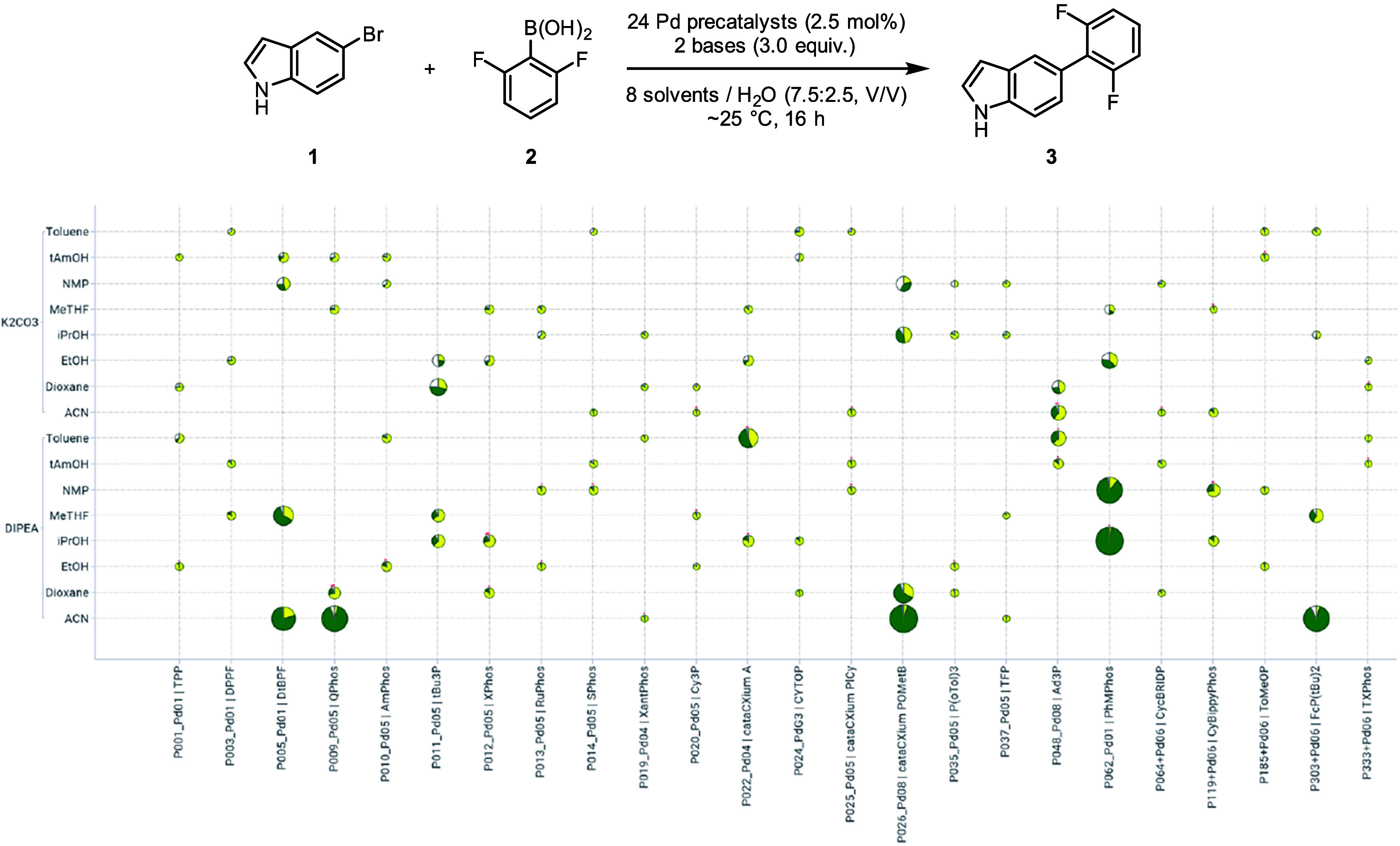
Frugal sampling results plotted against the full factorial matrix to assess the reaction between **1** and **2**. Pie charts were sized based on the amount of product. Pie sectors represent the calculated assay yields of the product (green) and the starting material (yellow). Reactions were performed on 15 μmol (3.0 mg) scale in 250 μL vials.^42^

In the follow-up screening, we completed the matrix of precatalyst combinations using acetonitrile and DIPEA to complement our initial findings (Figure 4). The results were consistent with the initial screening and allowed us to identify additional competent precatalysts (**P011_Pd05 | tBu**_**3**_ **P**, **P048_Pd08 | Ad**_**3**_ **P** or **P062_Pd01 | PhMPhos**) that delivered excellent yields. Notably, the pronounced reactivity of these precatalysts could have been inferred based on the frugal sampling results. Overall, the data revealed a strong influence of both the base and the ligand. However, further investigations would be required to fully understand the solvent trends.

**Figure 4:**
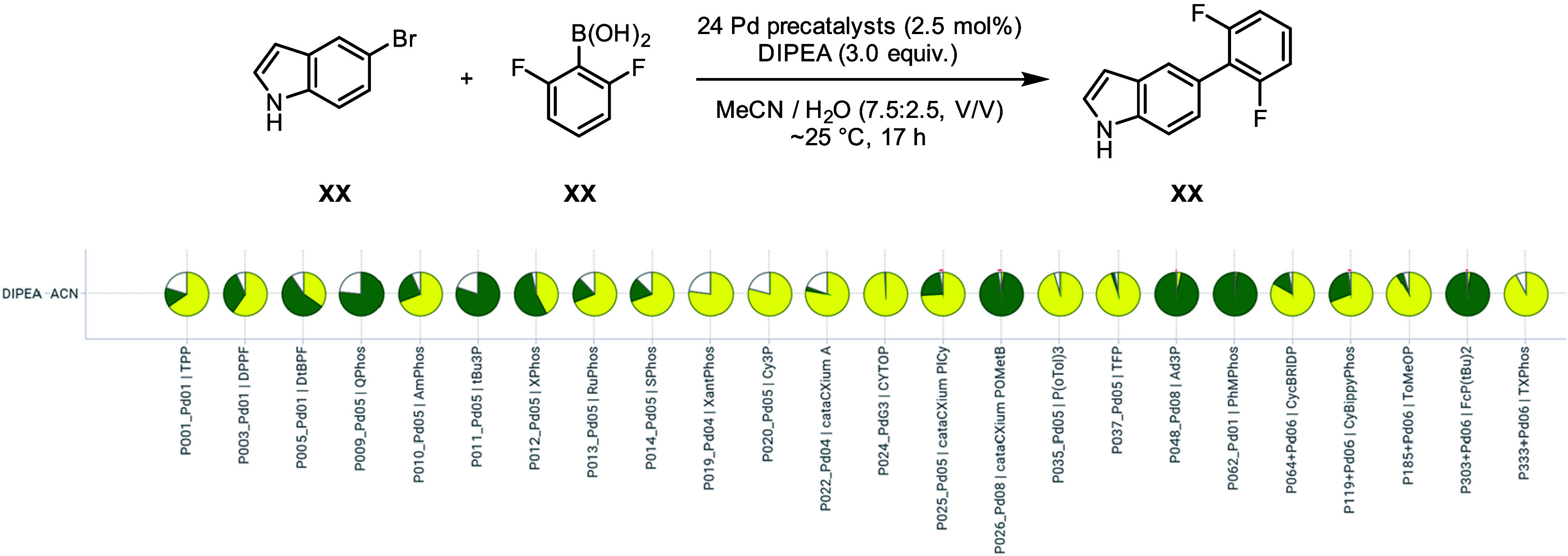
Follow-up screening using the best solvent-base combination found previously. Pie sectors represent the calculated assay yields of the product (green) and the starting material (yellow). Reactions were performed on 51 μmol (10 mg) scale in 1 mL vials.

For cost and supply chain considerations, our standard process optimization workflow involves replacing easily activated, IP-protected precatalysts with a combination of the corresponding ligand of interest and a readily available Pd source.^43^ As depicted in Figure 5, we selected four promising ligands (*t*Bu_3_P, cataCXium POMetB, Ad_3_P and FcP­(*t*Bu)_2_) identified through their ligand-Pd precomplexes for evaluation with five Pd sources (Pd­(OAc)_2_, Pd_2_(dba)_3,_ [Pd­(*t*Bu-Ind)­Cl]_2_, [Pd­(cinnamyl)­Cl]_2_ and [Pd­(αMeNAP)­Br]_2_). To better differentiate among the different Pd sources, the screening was performed at reduced loadings (down to 0.75 mol % Pd content, compared to 2.5 mol % in the initial screening). For comparison, the ligand-Pd precomplexes employed in the previous screenings (“Pd/L-Complex” column) are also included in the plate.^44^ Pleasingly, we found that two Pd sources ([Pd­(cinnamyl)­Cl]_2_ and [Pd­(αMeNAP)­Br]_2_) performed extremely well even at those lower Pd loadings with three out of four of the ligands investigated. Depending on project timelines and requirements, an initial scale-up with isolation studies could be conducted, or the reaction’s robustness may be further evaluated through a DoE.

**Figure 5:**
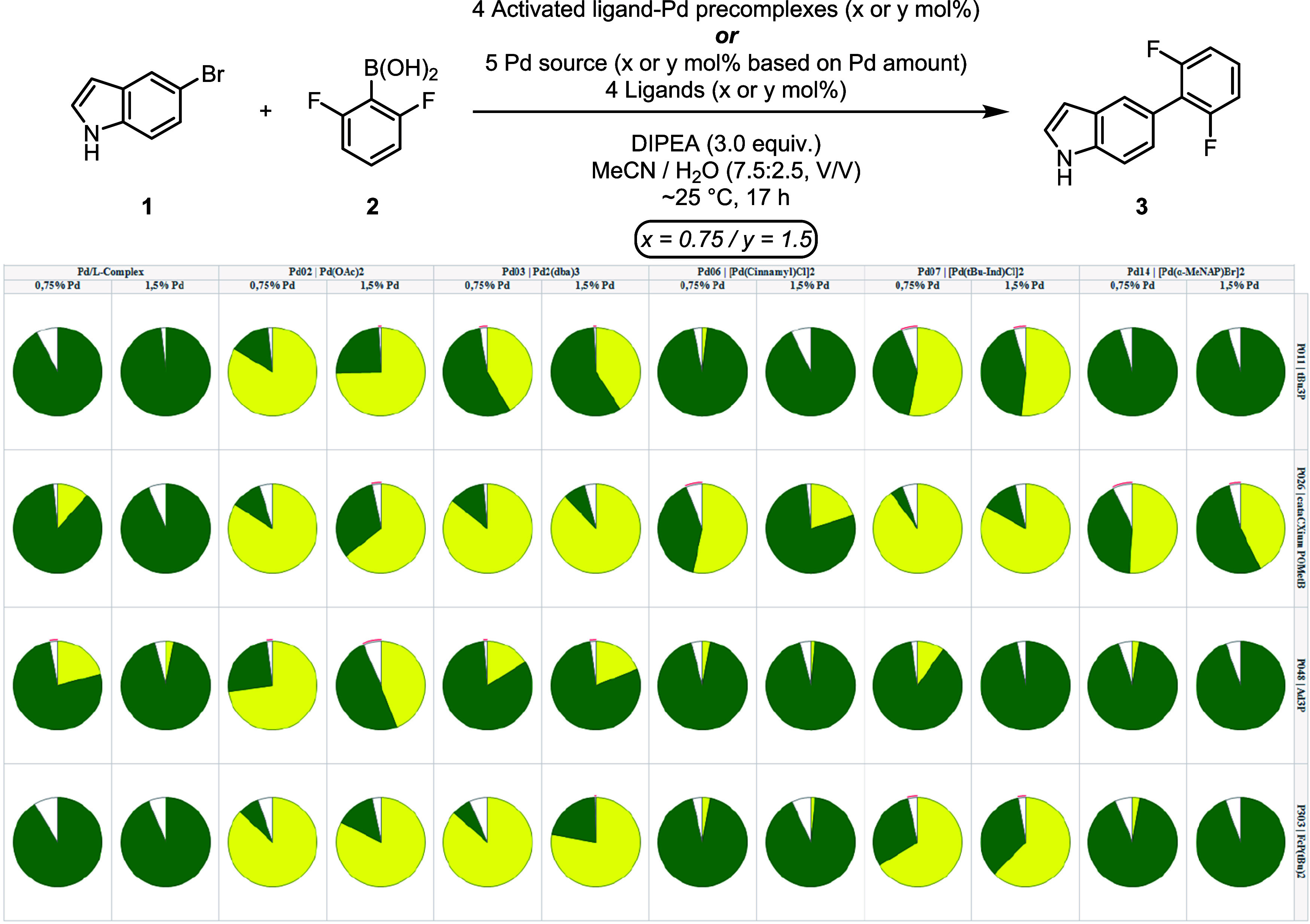
Identification of competent Pd sources with the most active ligands identified before. Pie sectors represent the calculated assay yields of the product (green) and the starting material (yellow). Reactions were performed on 15 μmol (3.0 mg) scale.

### C­(sp^2^)-C­(sp^3^) Suzuki-Miyaura Coupling

For the second example, we selected the cross coupling reaction between the highly sterically hindered aryl bromide **4** and cyclopropyl boronic acid (**5**).^45,46^ We opted for the same design as before (24 Pd precatalysts, 8 solvents and 2 bases) except that the screening was conducted at 80 °C (Figure 6). In this transformation, we observed that *i*PrOH and EtOH consistently led to significant formation of the protodehalogenated side-compound **7**. In contrast, satisfactory conversions and yields were achieved when toluene (or *t*AmOH) was used in combination with K_2_CO_3_.

**Figure 6:**
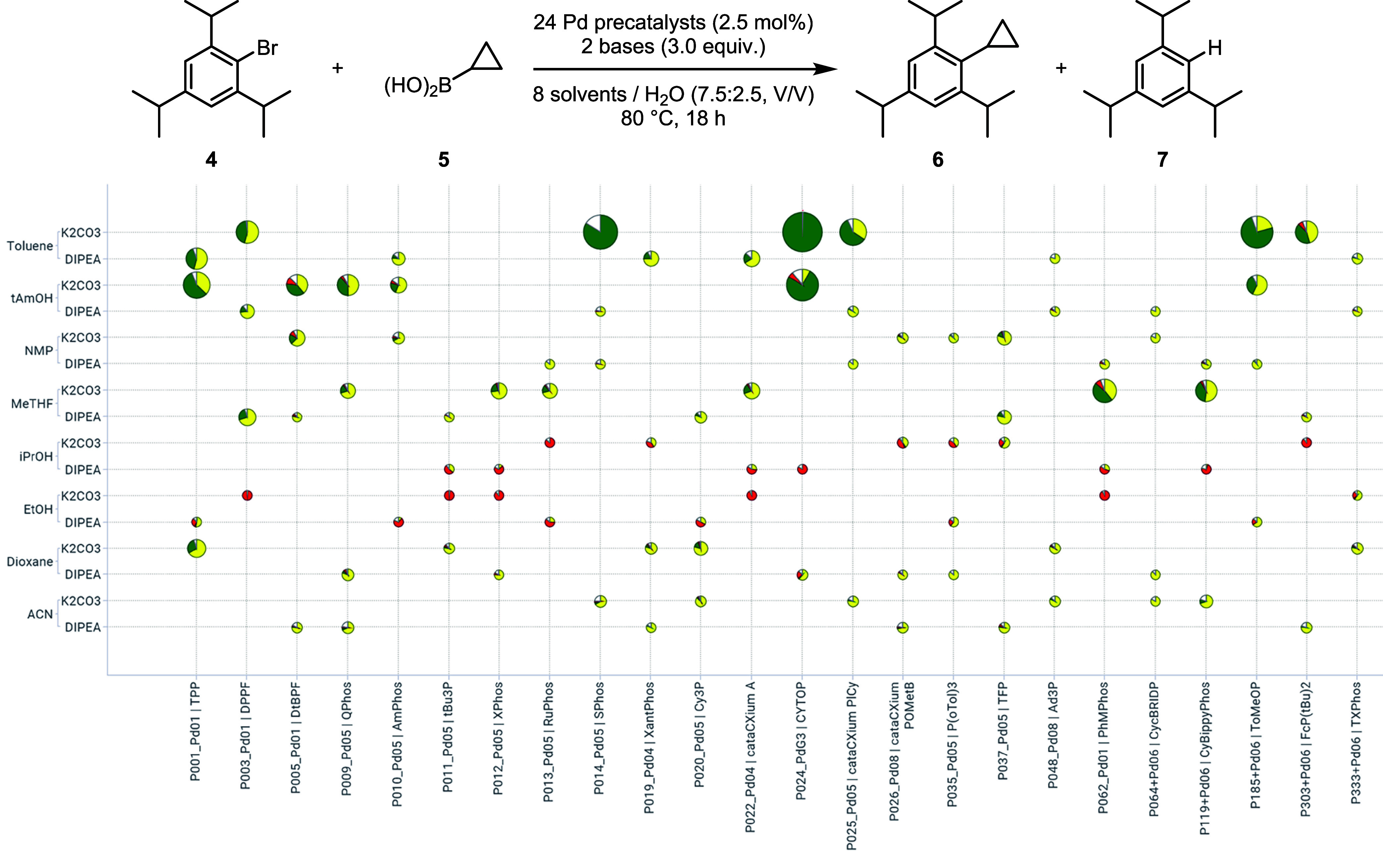
Frugal sampling results plotted against the full factorial matrix to assess the reaction between **4** and **5**. Pie charts were sized based on the amount of product. Pie sectors represent the calculated assay yields of the product (green), the starting material (yellow) and side-product (red). Reactions were performed on 34 μmol (9.6 mg) scale in 1 mL vials.^42^

As in the first case study, we evaluated the 24 precatalysts using the most promising base-solvent combination identified during the primary screening, namely K_2_CO_3_ and toluene (Figure 7). The observed trends were consistent with the previous screen, though small discrepancies in conversion and side-product formation were noted. To assess the reproducibility and gain a better understanding of the impact of continuous process parameters, we conducted a DoE investigation on a larger scale (100 mg per experiment) using the cost-effective PdCl_2_(PPh_3_)_2_ (**P001_Pd001**|TPP) at a reduced catalyst loading (1.5 mol %). We systematically varied the stoichiometry of **5**, the base and the H_2_O content, a parameter that can have critical importance in this type of cross coupling.^47^ The results at the four center points indicated good reproducibility at this scale (61% average; standard deviation = 3%), while the product yields ranged from 26 to 81% area percentages. The model revealed that a low H_2_O content (∼1 V) and a high amount of K_2_CO_3_ (∼3.0 equiv) significantly enhanced product formation (see Supporting Information for details; section 3g). This example underscores that minor variability can sometimes be inherent to HTE. However, its primary purpose is to reveal overarching trends rather than provide absolute values, which was achieved here with a relatively small number of experiments in comparison to the chemical space explored.

**Figure 7:**
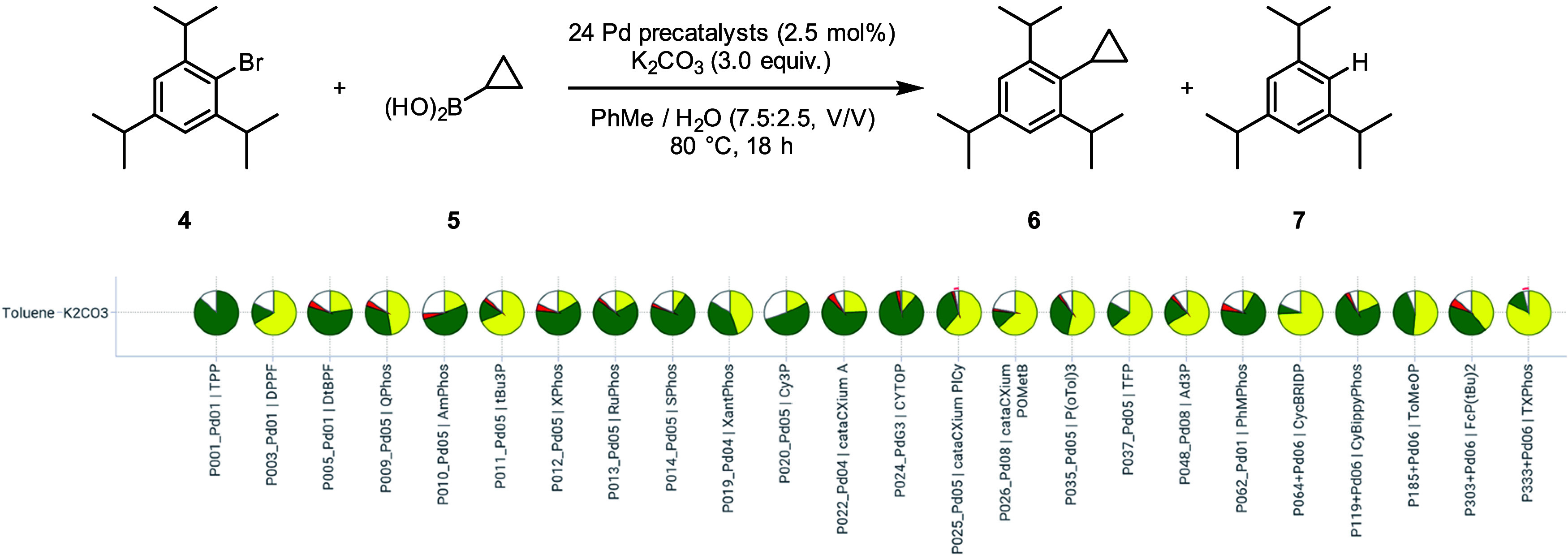
Follow-up screening using the most promising base-solvent combination with the 24 different precatalysts. Pie sectors represent the calculated assay yields of the product (green), the starting material (yellow) and side-product (red). Reactions were performed on 34 μmol (9.6 mg) scale in 1 mL vials.

### HetArCl – Azole Buchwald-Hartwig Amination

Having obtained promising results for both Suzuki-Miyaura cross couplings using the frugal sampling, we next turned our attention to the Buchwald-Hartwig amination. For the first C–N cross coupling reaction, we selected the challenging transformation between aryl chloride **8** and imidazole **9**, which had been well documented (Figure 8).^16,22,48^ This coupling is characterized by a limited number of active catalytic systems, rendering it particularly ambitious for a minimalist sampling approach. Compared to our standard conditions for azoles/amides couplings, we enlarged our standard set of precatalysts from 12 to 24 (see Supporting Information for details; section 4a), as well as the number of bases from two to four (NaOTMS, NaO*t*Am, K_3_PO_4_ and Cs_2_CO_3_) in combination with four solvents of different polarities (Toluene, *t*AmOH, NMP, MeTHF).^49^ Following the method described in Figure 2, in which solvents and bases were dispensed in a grid-like format, we used a custom script to assign the precatalysts to their respective wells (see additional spreadsheet). To maximize diversity in this plate design, we introduced the constraint that each precatalyst had to be used with a unique base and solvent combination. Notably, the frugal sampling is flexible enough and can accommodate varying numbers of parameters to adapt to various experimental requirements. Experimentally, we found that the trends were consistent with both our ultra-HTE and the pool-and-split reports.^16,22^ In general, strong bases were poorly tolerated, often leading to nonspecific reactivity, and only a limited number of precatalysts proved active. At this microscale loading, AlPhos (through its corresponding Pd precatalyst) yielded the best results, followed by the structurally related *t*BuBrettPhos and Me_4_ *t*BuXPhos. Overall, even for a complicated transformation, the frugal sampling enabled the identification of pertinent systems, which could be considered for further optimization.

**Figure 8:**
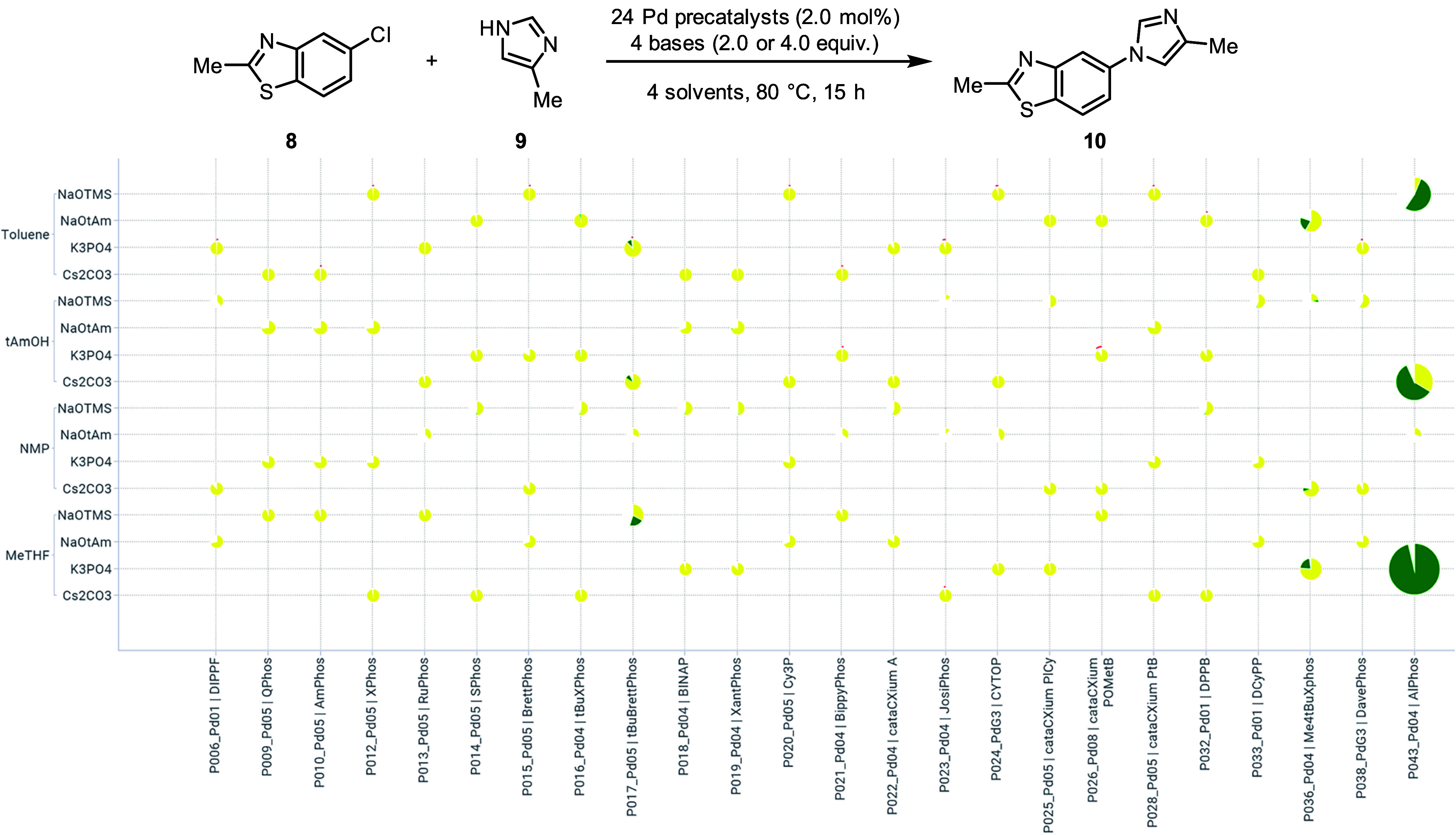
Frugal sampling results plotted against the full factorial matrix to assess the reaction between **8** and **9**. Pie charts were sized based on the amount of product. Pie sectors represent the assay yields of the product (green) and the starting material (yellow). Reactions were performed on 19 μmol (3.5 mg) scale in 250 μL vials.

### ArBr – Secondary Amine Buchwald-Hartwig Amination

For the final case study, we decided to investigate the coupling between bromo-4-*tert*-butylbenzene (**11**) and a sterically hindered secondary amine, such as 2-benzylpiperidine (**12**).^50^ For this example, we aimed to explore a broader chemical space by expanding the scope of the ligands tested (48 precatalysts, see Supporting Information; section 5a) in combination with four solvents (dioxane, MeTHF, *t*AmOH and toluene) and four bases (NaOTMS, NaO*t*Am, K_3_PO_4_ and Cs_2_CO_3_), resulting in a full factorial matrix of 768 options. To streamline the optimization workflow, we utilized SuntheticsML, a commercial platform that offers advanced sampling strategies, BO algorithms for experiment recommendations and data visualization tools. For the frugal sampling, we used first a quasi-random Sobol algorithm, which offered a more balanced distribution compared to the Latin hypercube sampling (LHS) option (see Supporting Information for a comparative analysis; section 5b), supplemented by a few manual adjustments to facilitate the solvent/base plating. Although it is less balanced compared to our uniform design, this alternative sampling method offers an alternative to the custom-script. Two 96-well MTPs were carried out in parallel (192 experiments, covering 25% of the total design space) using a small total amount of starting material (1.0 mg of **11**/well). We were pleased to find that several catalytic systems delivered high conversions and yields, although no distinct trends for solvents and bases could be recognized at this stage (Figure 9).

**Figure 9:**
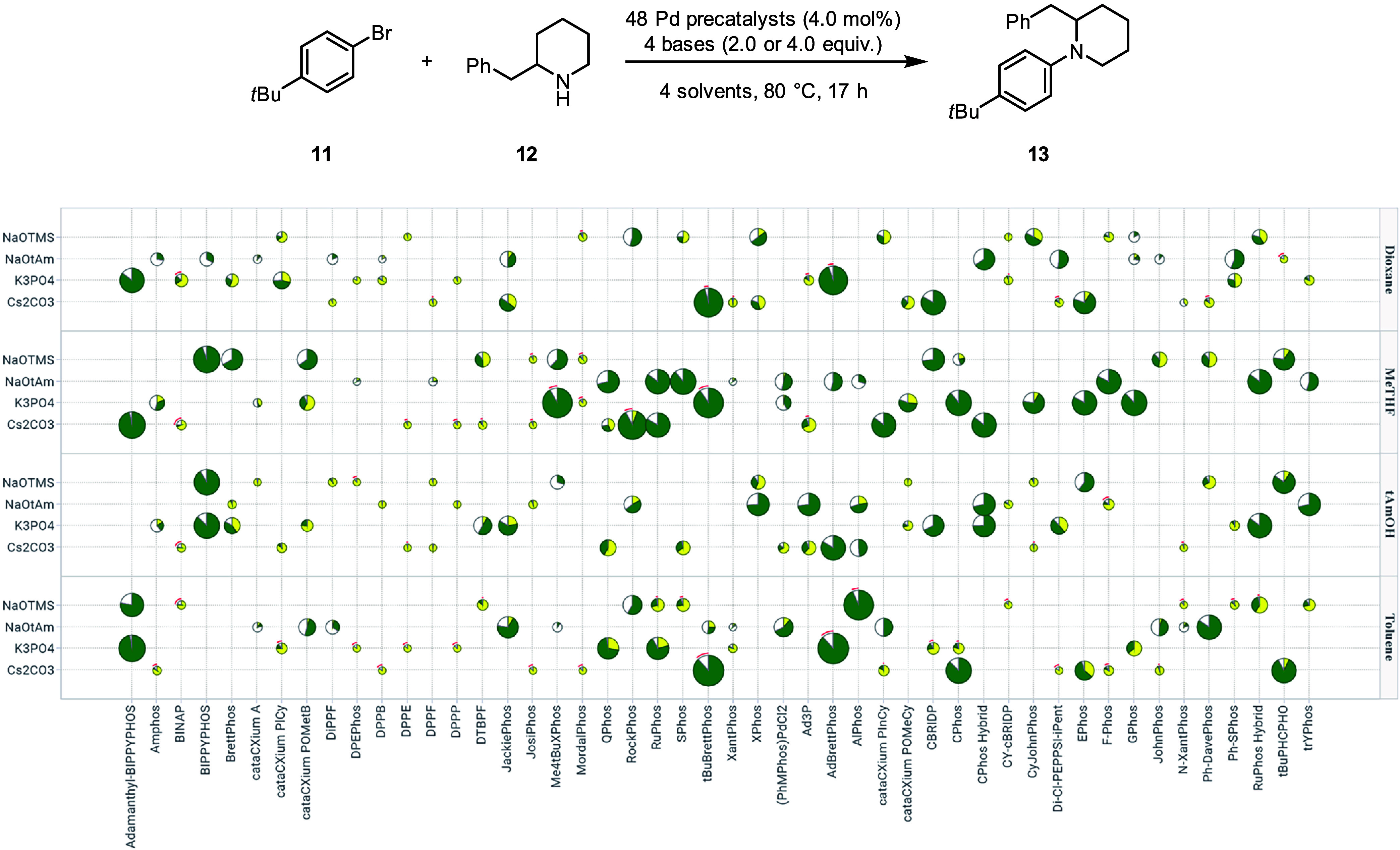
Frugal sampling results plotted against the full factorial matrix to assess the reaction between **11** and **12**. Pie charts were sized based on the amount of product. Pie sectors represent the calculated assay yields of the product (green) and the starting material (yellow). To improve readability, only the ligand of interest from the precatalyst was displayed on the *x*-axis. Reactions were performed on 4.7 μmol (1.0 mg) scale in 250 μL vials.^42^

Although the screen yielded combinations that already achieved a product area percentage of up to 100%, smooth delivery of preclinical and early clinical drug substance hinges not only on reaction performance but also on cost of goods. We hypothesized that improvements could be made to both categorical (e.g., employing inexpensive ligands) and continuous (e.g., reducing reagent stoichiometries) factors to achieve a more cost-effective transformation. Recently, Sin and Schwaller demonstrated that 96-well HTE optimization campaigns can be effectively integrated with BO approaches to navigate high-dimensional search spaces and optimize related transformations.^51^ While highly effective, their methodology can be technically challenging to implement, requiring nearly 96 independent reaction conditions and is limited to categorical factors, with temperature evaluated only as a discrete numerical variable. Inspired by their report, we initially considered the frugal sampling data set to initialize a Bayesian surrogate model using SuntheticsML (see Supporting Information; section 5e). However, we anticipated that the limited diversity in continuous variable values could introduce bias into the design space exploration. Instead, we designed a stepwise approach: first leveraging the frugal sampling to identify the most promising combinations of categorical parameters, followed by BO to refine the reaction conditions. The BO protocols would explore both continuous and categorical parameters and be executed using a smaller batch size of 24 experiments. This number was considered easily attainable without the need for sophisticated robotic instrumentation. Additionally, we decided to increase the reaction scale compared to the previous screen (10 mg/well instead of 1 mg) to minimize experimental variability.

Leveraging our 192 experiments, we prioritized 12 ligands that combined low cost with high activity of their corresponding precatalysts (Figure 10 – Step A; see Supporting Information for details).^52^ This was achieved through a cost-penalized yield obtained by dividing the reaction yield by the logarithm of the ligand’s price. Given the substantial difference in ligand prices, this metric effectively penalized more expensive ligands while maintaining a reasonable range for the adjusted yields. We then applied a LHS to evaluate both categorical (precatalysts, solvents and bases) and continuous (precatalyst loading, amounts of base and **12**, dilution) factors across 24 experiments (Figure 10 – Step B). Similar to the frugal sampling screen, we obtained reaction conditions that delivered high yields, but without reaching our cost optimization objective.

**Figure 10:**
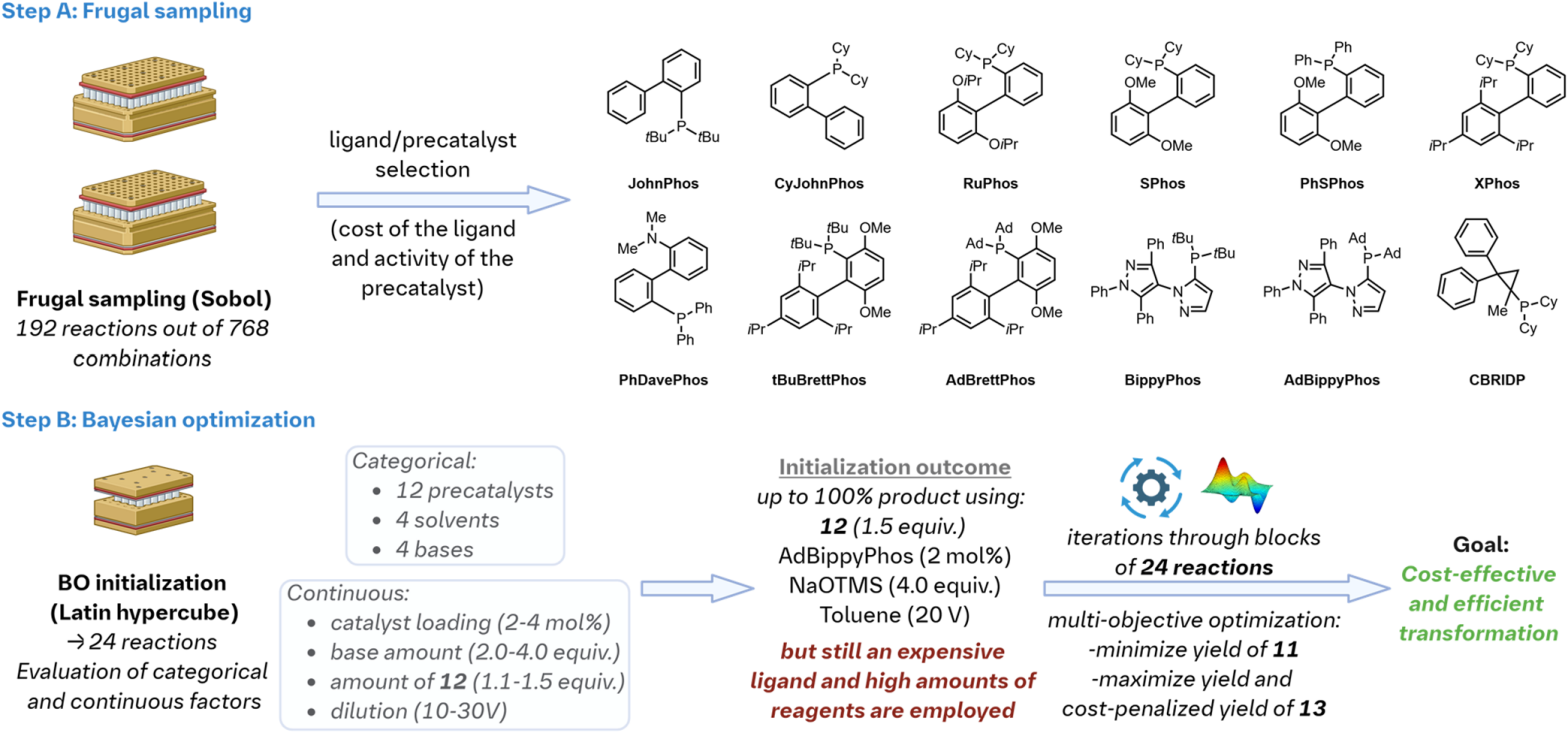
Summary of the workflow to optimize the coupling between **11** and **12**. The figure was created in Biorender.^13^

For the first BO iteration, the multiobjective optimization was aimed at maximizing both the yield and its cost-penalized counterpart, while simultaneously minimizing the residual starting material (**11**).^53–55^ Regarding the featurization of the categorical factors, we opted for SMILES-derived Chemical Encodings using Sunthetics’ proprietary workflow for chemical variables, which supported the parametrization of precatalysts, solvents and bases. In our experience, this parametrization workflow has outperformed traditional categorical encodings like one-hot encodings and proven to yield better models. Based on the LHS initialization results, the acquisition function mostly focused on readily available ligands (e.g., PhSPhos and RuPhos) and strong bases (NaO*t*Am and NaOTMS). Experimentally, a practical batch size of 24 conditions was executed (18 exploitation and 6 exploration conditions) yielding transformations with excellent yields (e.g, SPhos, 2.0 mol %; NaO*t*Am, 2.0 equiv.; 98%) and, on average, outperformed the initialization batch. For the second iteration, we ran ad hoc adjustments of the continuous parameters, narrowing the precatalyst loading (from 2–4 mol % to 0.5–2 mol %) and the base amount (from 2.0–4.0 equiv. to 1.2–4.0 equiv), with the goal of obtaining more economical combinations. In the exploitation experiment proposals, the model recommended greater ligand diversity and to employ mostly NaO*t*Am typically in higher quantities (>2.0 equiv). Solvent choice had minimal impact on the transformation, whereas more concentrated reaction mixtures seemed to be beneficial. After performing the recommended reactions, the average yield improved further, even with reduced precatalyst loading. For the third iteration, the cost estimation was refined to further optimize reagent stoichiometry by recalculating the penalized yields using eq 1. In this equation, we incorporated the cost of the base (NaO*t*Am) and we postulated that the ligand-Pd precomplex could be substituted (cf. Figure 5) with its corresponding ligand and a commonly used Pd source, such as [Pd­(cinnamyl)­Cl]_2_.

$$\mathrm{Approximate}\;\mathrm{cost}=\left[\left(\mathrm{cost}\;\mathrm{of}\;\mathrm{the}\;\mathrm{ligand}\right)+1/2\left(\mathrm{cost}\;\mathrm{of}\;{\left[\mathrm{Pd}\left(\mathrm{cinnamyl}\right)\mathrm{Cl}\right]}_{2}\right)\right]\times \mathrm{cat}.\mathrm{loading}+\left(\mathrm{cost}\;\mathrm{of}\;\mathrm{NaO}t\mathrm{Am}\right)\times \mathrm{equiv}.\;\mathrm{of}\;\mathrm{base}$$ 1

Upon executing the suggested batch of 24 experiments, we were pleased to ascertain that over 60% of the tested conditions yielded product area percentages exceeding 95%. We found that RuPhos and PhDavePhos (as ligand-Pd precomplexes) performed extremely well at low loadings (down to 0.5 mol %) in the presence of NaO*t*Am (>1.5 equiv), regardless of the solvent employed and under relatively concentrated mixtures (down to 10 V). Ultimately, the iterative optimization process proved effective in enhancing product formation while simultaneously reducing the costs (Figure 11). The algorithm’s stepwise decision-making was further elucidated through partial dependence plots that depict the relationships between the variables (see Supporting Information; section 5e).

**Figure 11:**
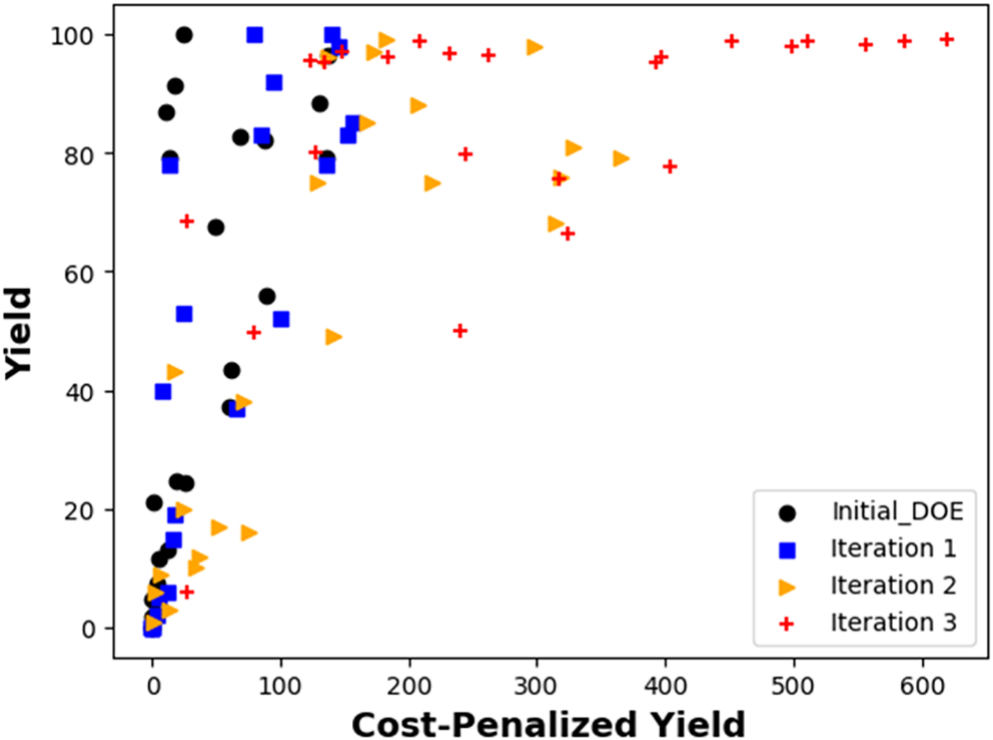
Evolution of the yield (as area percentages) and cost-penalized yield over the different iterations using eq 1. “Initial DOE” refers here to the 24 experiments performed to initialize the BO algorithm.

Having reduced the dimensionality of the optimization problem, we set to refine our understanding of the reaction using more homogeneous and space-filling experimental designs. As suggested by Braconi and Godineau,^56^ the combination of BO with DoE offers a promising combination, where BO facilitates global exploration, and DoE enables local optimization. Following a central composite design, we studied the influence of the stoichiometry of the base (NaO*t*Am) and the amine starting material **12** in more detail, using PhDavePhos (as its corresponding ligand-Pd precomplex) at 0.5 mol % in toluene (10 V). PhDavePhos Pd precomplex was chosen due to the cost of its ligand being less expensive compared to RuPhos.^52^ In the case of toluene, it was selected based on its prior use at the same dilution with this precatalyst in the third BO iteration, where an excellent yield of **13** was obtained (99%). After carrying out the ten experiments, we were pleased to find that the transformation proved to be extremely robust. It tolerated reduced amounts of base and amine without impacting the yield or conversion, which was quantitative across the evaluated design space (see Supporting Information for details; section 5f). The combination of BO and DoE allowed us to identify a rarely used, cost-effective ligand, a reduction in precatalyst, base and amine stoichiometries, while allowing the reaction to be performed at higher concentration in toluene.

## Conclusion

In summary, reflecting on over a decade of HTE experimentation and optimization campaigns at Boehringer Ingelheim, we noticed an intrinsic redundancy in the results of the usual full factorial, grid-like HTE screening plates. Based on this observation, we introduced novel sampling strategies for screening a broad set of parameters for complex catalytic transformations using a minimal number of experiments. These approaches hinge on executing only a fraction of all possible combinations (25%) through frugal, quasi-random samplings of the reaction space, while relying on standard 96-well MTP and HTE technology to perform the screenings. We demonstrated that, for four different cross coupling reactions, the first-pass frugal screens (evaluating up to 48 different precatalysts, four bases and four solvents in parallel) retain sufficient information to guide subsequent optimization steps. These include identifying an effective yet affordable catalytic system, DoE-based parameter fine-tuning or cost-aware BO protocols. The frugal samplings enhance the exploratory power of HTE beyond the traditional array design and are now routinely applied in our pipeline projects. To further contribute to the growth of HTE, we provide the sampling strategies through the plate designs and a customizable spreadsheet to fit the needs of chemists.

## Supplementary Material





## Data Availability

Spreadsheet for personalizing the plate designs is available at 10.6084/m9.figshare.31124158 (https://figshare.com/s/b7b8c196609e3820f372)

## Notes

Bayesian optimization results (XLSX)
Additional details on the design of frugal sampling approaches, guidelines, comparison with other sampling methods, and experimental procedures (PDF)

## Abbreviations

BO: Bayesian optimization
DIPEA: *N*,*N*-diisopropylethylamine
DoE: Design of Experiments
HTE: high-throughput experimentation
LHS: Latin hypercube sampling
MeTHF: 2-methyltetrahydrofuran
MTP: microtiter plate
NMP: *N*-methyl-pyrrolidone
