# Supplementary material for: Frugal Sampling Strategies for Navigating Complex Reaction Spaces
Source: Org Process Res Dev. 2026 Apr 10;30(5):1279–89. doi: 10.1021/acs.oprd.6c00027 (PMC13186068; doi:10.1021/acs.oprd.6c00027)
Supplement: Supplementary file 2 [file op6c00027_si_002.pdf]

Supplementary Information

Frugal Sampling Strategies for Navigating Complex Reaction Spaces

Vincent Porte[a], Luca Hepp[a], Philipp Kollmus[a], Shizhao Lu [b*], Eloisa Serrano[a], Daniela Blanco[b] and Marco Santagostino[a]**

[a]Chemical Development Germany, Boehringer Ingelheim Pharma GmbH & Co. KG, Biberach an der Riß, 88397 Germany

[b]Sunthetics, Inc, 3055 Hunter Rd, San Marcos, 78666, TX, USA

Vincent Porte: porte.vincent@proton.me

Shizhao Lu: giselle@sunthetics.io

Marco Santagostino: marco.santagostino@boehringer-ingelheim.com

Table of contents

1. General Information 4

a. Glassware and reagents: 4

b. ChemBeads 4

c. Screening: 4

d. HPLC method: 5

e. Data visualization 5

f. Bayesian optimization (BO) 5

2. Frugal sampling 6

a. Distributing 2 bases x 8 solvents x 24 precatalysts into a 96-well MTP 6

b. Distributing 4 bases x 4 solvents x 24 precatalysts into a 96-well MTP 7

c. Comparison between the frugal sampling approach with Design of Experiments (DoE) and Bayesian optimization (BO) 8

d. Guidelines 8

3. Suzuki-Miyaura cross couplings 9

a. Pd precatalysts employed for the formation of 3 and 6. 9

b. Frugal sampling for the formation of 3 11

c. Secondary screening for the formation of 3 12

d. Screening of Pd sources for the formation of 3 13

e. Frugal sampling for the formation of 6 15

f. Secondary screening for the formation of 6 16

g. DoE for the formation of 6 17

4. Buchwald-Hartwig – coupling between 8 and 9 19

a. Pd precatalysts 19

b. Frugal sampling for the formation of 10 20

5. Buchwald-Hartwig – coupling between 11 and 12 21

a. Pd precatalysts 21

b. Comparison between Sobol and Latin hypercube samplings 23

c. Frugal sampling for the formation of 13 25

d. General procedure for the LHS sampling and BO experiments – synthesis of 13 26

e. Bayesian optimization visualization 27

i. Using data from the frugal sampling (192 experiments) 27

ii. Partial dependence plots 29

f. DoE for the formation of 13 31

g. **Isolation and characterization of 13** 33

1. General Information

a. Glassware and reagents:

Unless otherwise stated, all glassware was used without prior drying and all reactions were performed under an atmosphere of nitrogen. All anhydrous, sure-seal quality solvents and reagents were used as received from commercial suppliers unless otherwise stated. The precatalysts, ligands and ChemBeads were stored in a desiccator filled with nitrogen or in a fridge.

b. ChemBeads

Glass beads (soda lime, typically 0.1 mm diameter) were purchased from BioSpec Products (https://biospec.com/products/glass-beads)

The precatalyst (52 µmol) was added in a vial containing glass beads (typically 2.6 g). An additional portion of glass beads (2.6 g, 100 µm) was charged such that the substance is "sandwiched" between the beads. The vial was submitted to ResonantAcoustic™ Mixing (RAM), 10 s at 40 g followed by 2 min at 80 g, resulting in the desired ChemBeads (10 µmol/g).

Procedure with premilling if non-homogeneous ChemBeads were obtained previously: In a 20-mL HDPE scintillation vial was added the desired substance (52 µmol) followed by the addition of 12-15 Zr balls. The vial was submitted to ResonantAcoustic™ Mixing (RAM), 10 s at 40 g followed by 2 min at 80 g. Glass beads (100 µm) were then added to the resulting finely ground powder (typically 5.2 g) and the vial was submitted to RAM, 10 s at 40 g followed by 2 min at 80 g, resulting in the desired ChemBeads (10 µmol/g).

c. Screening:

Solid chemicals were dosed using an in-house build system or with an Unchained Lab Junior. Liquid chemicals or solutions were dosed using electronic multi-channel or single-channel pipettes inside the glovebox.

In our primary screenings, ligands are assessed either as preformed Pd(II) precatalysts (**PXXX_PdXX**) or, more seldom, as physical mixtures of the ligand with a competent Pd source such as $[Pd(cinnamyl)Cl]_2$ (**PXXX+PdXX**), when the corresponding precomplex is not readily available.

Undesired solvent (CH_2Cl_2 or THF) used for the stock solutions of the starting materials was removed using a Genevac vacuum centrifuge or by slow evaporation under a N_2 flux.

Reaction screenings were carried out in 1.0 mL vials (8 x 30 mm) or 250 µL vials (5 x 31 mm) purchased from Analytical Sales and Services (84001-CASE and 20303-CASE). Below the reactors was a fluorosilicone rubber mat. Directly above the tops of glass vial reactors was a perfluoroalkoxy sealing film and above that was another 3.175 mm thick fluorosilicone rubber sealing mat.

HPLC assay yields were calculated using an internal standard (biphenyl). If the reference material was unavailable, a reaction giving full conversion and a clean HPLC trace was used to set the yield at 100%.

d. HPLC method:

Table S1: Characteristic of the HPLC method employed.

HPLC method	Method 1
Wavelength	230 nm
Temperature	60 °C
Column	Kinetex C18 2.6 µm 2.1 x 30mm
Solvents	A: 0,2 % HCOOH
	B: MeCN
Flow	2.5 mL/min
Gradient	0.03' 3 %B
	0.5 100 %B
	0.55' 100 %B
	0.6' 3 %B
Stoptime	0.65 min

e. Data visualization

Data visualization was performed using Spotfire^{TM}. The size of each pie sector indicates the relative amount of the reaction components. In some cases, the pie charts are sized by the amount of product measured; the bigger the size, the more product.

f. Bayesian optimization (BO)

In the current study, Chemical Encoding that uses SMILES to create high-dimensional, chemically-informed encodings of ligands, solvents and bases were employed. For other numerical variables, no encodings were necessary. The acquisition functions used in SuntheticsML's BO algorithm give exploitation points to experiments that maximize the expected improvement of the outputs and give exploratory points to experiments that minimize the prediction uncertainty metrics such as standard deviation.

2. Frugal sampling

The proposed sampling algorithms were designed to conduct complex screenings in 96-well format by carefully considering the requirements for practical plating, especially for bases and solvents. Spreadsheet for personalizing the plate designs is available at 10.6084/m9.figshare.31124158 (https://figshare.com/s/b7b8c196609e3820f372).

a. Distributing 2 bases x 8 solvents x 24 precatalysts into a 96-well MTP

A set of 24 precatalysts was randomized and distributed across quadrant A (see Table S2). Following a second randomization step to reduce potential bias, quadrant B was filled. Quadrant C and D were then filled using a brute-forced method (guess, test, repeat) to ensure that no precatalyst was exposed twice to the same solvent (Solv#). In other words, no precatalyst appears twice within the same solvent row. In the final plate design, each quadrant contains all 24 precatalysts. Each base contains two instances of each precatalyst. No precatalyst appears more than once in a solvent row.

Table S2: Partitioning of the 96-well MTP for assigning the 24 precatalysts while maintaining practicality for two bases and eight solvents.

	**Base1**	**Base2**
**Solv1**	Quadrant A	Quadrant C
**Solv2**		
**Solv3**		
**Solv4**		
**Solv5**	Quadrant B	Quadrant D
**Solv6**		
**Solv7**		
**Solv8**		

b. Distributing 4 bases x 4 solvents x 24 precatalysts into a 96-well MTP

A set of 24 precatalysts was randomized and distributed across quadrant A (see Table S3). Quadrant B was then filled using a brute-force method (guess, test, repeat) to ensure that no duplicates occurred within any solvent rows. The set was randomized again, and quadrant C was filled. Afterwards, the rows were checked for duplicates. If a duplicate was found, it was removed and replaced with new guesses from the remaining precatalysts. This process was repeated until no duplicates remained, resulting in 18 precatalysts being assigned to each solvent row. Quadrant D was then filled by placing the six remaining precatalysts that had not yet been used in the corresponding solvent row. In the final plate design, each quadrant and each solvent row contains all 24 precatalysts. Each precatalyst appears once in each base, and no precatalyst appears more than once in a solvent row.

Table S3: Partitioning of the 96-well MTP for assigning the 24 precatalysts while maintaining practicality for four bases and four solvents.

	Base1	Base2	Base3	Base4
Solv1	Quadrant A	Quadrant B	Quadrant C	Quadrant D
Solv2				
Solv3				
Solv4				

As illustrated in Figure S1, the plate design used for achieving the balanced distribution (4 rows and 24 columns) is then reorganized to a conventional 96-well MTP (8 rows and 12 columns).

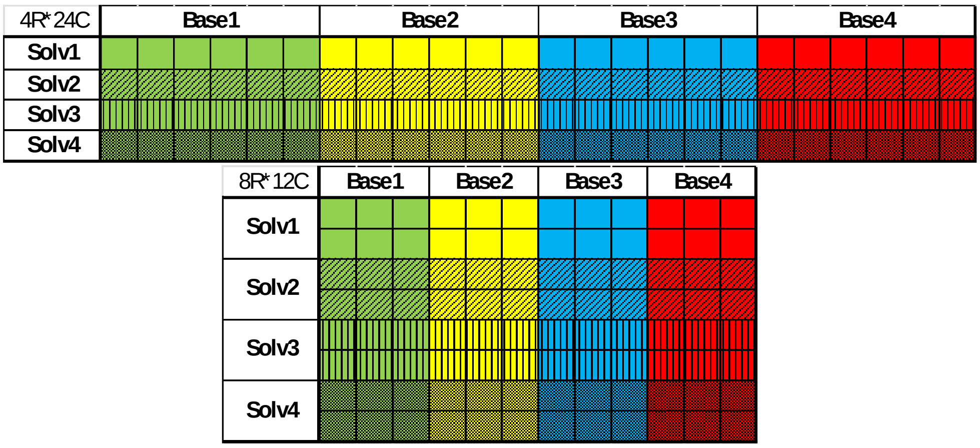

Figure S1: Reorganization of Table S3 (4 rows and 24 columns) into a 96-well MTP (8 rows and 12 columns). The colors and patterns only illustrate the plate layout reorganization and do not contain information related to the precatalysts.

c. Comparison between the frugal sampling approach with Design of Experiments (DoE) and Bayesian optimization (BO)

If we consider a scenario of 24 precatalysts x 8 solvents x 2 bases:

A full factorial design and a grid-like layout (DoE / HTE) would require 384 experiments. For a DoE using an optimal (combined) design, without any replicate points and lack-of-fit points, 223 experiments would be required. Our custom script would lead to only 96 experiments.

For a BO, we believe this type of assessment (i.e. number of experiments) is difficult to generalize as it depends on the transformation. In addition, the total number of experiments will depend on the initialization protocol employed and the number of reactions that can be performed in each iteration. If only a small number of experiments can be performed in each campaign, identifying the categorical parameters could be time-consuming.

d. Guidelines

Compared with other sampling methods that efficiently explore the chemical space (e.g., Sobol and Latin hypercube), the frugal sampling, through its design, minimizes redundancy and offers a plate design that can be easily conducted within a 96-well MTP. Based on our experience, we believe that the frugal sampling is currently well-suited for a full factorial space of 384 combinations (or multiple) which is then “compressed” within a 96-well MTP (or multiple). However, we do not exclude other scenarios where the frugal sampling could also apply.

For additional transformations related to pipeline projects, which are not reported in this manuscript for IP reasons, the frugal samplings have consistently revealed clear overarching trends. We believe that the frugal sampling approaches can accelerate the identification of relevant trends due to its important exploratory nature and non-iterative character. Additionally, unlike optimal designs and BO, the implementation of our frugal sampling approaches is greatly facilitated by the grid-like plating of some of the categorical variables.

In the absence of positive hits or, conversely, an overabundance of hits indicates that the transformation is either exceptionally demanding or trivial, respectively. In such cases, alternative optimization strategies should be considered.

3. Suzuki-Miyaura cross couplings

a. Pd precatalysts employed for the formation of 3 and 6.

Table S4: Selection of the Pd precatalysts for Suzuki-Miyaura cross coupling reactions. [a] Physical mixture of [Pd(cinnamyl)Cl]$_2$ and the ligand; CAS N° of the ligand and MW of the mixture are displayed.

Entry	Internal Acronym	CAS N°	MW (g/mol)	Name
1	P001_Pd00 | TPP	13965-03-2	701,9	Bis(triphenylphosphine)palladium(II) dichloride
2	P003_Pd01 | DPPF	138549-82-3	760,7	[1,1′-Bis(diphenylphosphino)ferrocene]dichloropalladium(II)
3	P005_Pd01 | DtBPF	95408-45-0	651,8	1,1′-Bis-(di-tert.-butylphosphino-)ferrocen-palladiumdichloride
4	P009_Pd05 | QPhos	1252598-33-6	907,7	Chloro(crotyl)[1,2,3,4,5-pentaphenyl-1'-(di-tert-butylphosphino)ferrocene]palladium(II)
5	P010_Pd05 | AmPhos	1334497-06-1	462,4	Chloro(crotyl)[di-tert-butyl(4-dimethylaminophenyl)phosphine]palladium(II)
6	P011_Pd05 | tBu3P	1334497-00-5	399,3	Chloro(crotyl)(tri-tert-butylphosphine)palladium(II)
7	P012_Pd05 | XPhos	1798782-02-1	673,7	Chloro(crotyl)(2-dicyclohexylphosphino-2',4',6'-triisopropyl-1,1'-biphenyl) palladium(II)
8	P013_Pd05 | RuPhos	1798781-96-0	663,6	Pd(crotyl)Cl;Chloro(crotyl)[2-Dicyclohexylphosphino-2',6'-di-i-sopropoxy-1,1'-biphenyl]palladium(II)
9	P014_Pd05 | SPhos	1798781-99-3	607,5	[(1,2,3-η)-2-Buten-1-yl]chloro[dicyclohexyl(2',6'-dimethoxy[1,1'-biphenyl]-2-yl)phosphine-κP]palladium
10	P019_Pd04 | XantPhos	879689-28-8	761,6	Palladium, chloro[(9,9-dimethyl-9H-xanthene-4,5-diyl)bis[diphenylphosphine-κP]](η3-2-propenyl)-
11	P020_Pd05 | Cy3P	307494-95-7	477,4	[(1,2,3-η)-2-Butenyl]chloro(tricyclohexylphosphine)palladium
12	P022_Pd04 | cataCXium A	2703751-81-7	541,5	Pd(Ad$_2$P(n-Bu))(allyl)Cl
13	P024_PdG3 | CYTOP	1350851-22-7	602,4	Chloro[(1,3,5,7-tetramethyl-5-phenyl-2,4,8-trioxa-6-phosphaadamantane)-2-(2-aminobiphenyl)]palladium(II)
14	P025_Pd05 | cataCXium PICy	n.a.	692,1	cataCXium PICy Pd(crotyl)OTf

15	P026_Pd08 | cataCXium POMetB	n.a.	730,2	(2-acetamidophenyl)(di-tert-butyl(1-(2-methoxyphenyl)-1H-pyrrol-2-yl)-l5-phosphaneyl)palladium, 4-methylbenzenesulfonate salt
16	P035_Pd05 | P(oTol)3	1385042-42-1	501,3	[(1,2,3-η)-2-Buten-1-yl]chloro[tris(2-methylphenyl)phosphine]-Palladium
17	P037_Pd05 | TFP	n.a.	430,2	Tri(2-furyl)phosphine Pd(crotyl)Cl
18	P048_Pd08 | Ad3P	1926980-77-9	848,4	[2-[(Acetyl-κO)amino]phenyl-κC][tris(tricyclo[3.3.1.13,7]dec-1-yl)phosphine]palladium 4-methylbenzenesulfonate
19	P062_Pd01 | PhMPhos	2768152-19-6	848,0	1-Diadamantylphosphino-1′-diphenylphosphinoferrocene palladium dichloride
20	P064+Pd06 | CycBRIDP[a]	1023330-38-2	663,6	Dicyclohexyl-(2,2-diphenyl-1-methyl-1-cyclopropyl)-phosphin +1/2 $[Pd(cinnamyl)Cl]_2$
21	P119+Pd06 | CyBippyPhos[a]	1021176-69-1	817,8	5-(Dicyclohexylphosphino)-1′,3′,5′-triphenyl-1′H-[1,4′]bipyrazole+1/2$[Pd(cinnamyl)Cl]_2$
22	P185+Pd06 | ToMeOP[a]	4731-65-1	611,4	Tris-(o-methoxyphenyl)-phosphin+1/2$[Pd(cinnamyl)Cl]_2$
23	P303+Pd06 | FcP(tBu)2[a]	223655-16-1	589,3	Di-tert-butylphosphinoferrocene+1/2$[Pd(cinnamyl)Cl]_2$
24	P333+Pd06 | TXPhos[a]	2376379-81-4	938,1	Dicyclohexyl[2,2'',4,4'',6,6''-hexakis(1-methylethyl)[1,1':3',1''-terphenyl]-2'-yl]phosphine+1/2$[Pd(cinnamyl)Cl]_2$

b. Frugal sampling for the formation of 3

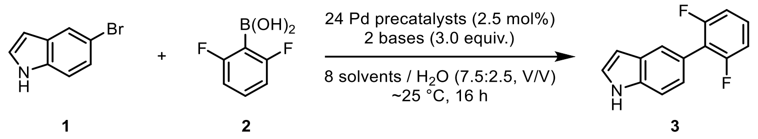

5-bromoindole (3.0 mg, 15 µmol, 1.0 equiv.) and 2,6-difluorophenylboronic acid (3.0 mg, 18 µmol, 1.2 equiv.) were dispensed as stock solutions in THF into a Paradox 96-well microtiter plate (MTP) filled with micro vials. The solvent was removed under a nitrogen stream. Pd precatalysts on ChemBeads (10 µmol/g, 2.5 mol%) were added sequentially to the appropriate vials.

*Table S5: Plate design (primary screening) for the coupling between **1** and **2**. For the Pd precatalysts, only the corresponding ligands are displayed.*

	**K2CO3**						**DIPEA**					
**EtOH**	tBu3P	cataCXium A	XPhos	PhMPhos	DPPF	TXPhos	TPP	RuPhos	Cy3P	ToMeOP	P(oTol)3	AmPhos
**iPrOH**	FcP(tBu)2	RuPhos	P(oTol)3	TFP	XantPhos	cataCXium POMetB	PhMPhos	CYTOP	tBu3P	CyBippyPhos	XPhos	cataCXium A
**tAmOH**	QPhos	TPP	DtBPF	ToMeOP	AmPhos	CYTOP	TXPhos	cataCXium PICy	Ad3P	SPhos	CycBRIDP	DPPF
**ACN**	Cy3P	cataCXium PICy	CyBippyPhos	SPhos	CycBRIDP	Ad3P	XantPhos	TFP	QPhos	cataCXium POMetB	FcP(tBu)2	DtBPF
**NMP**	DtBPF	cataCXium POMetB	TFP	AmPhos	P(oTol)3	CycBRIDP	cataCXium PICy	RuPhos	SPhos	ToMeOP	PhMPhos	CyBippyPhos
**Dioxane**	Cy3P	tBu3P	XantPhos	TXPhos	Ad3P	TPP	P(oTol)3	CycBRIDP	QPhos	XPhos	cataCXium POMetB	CYTOP
**MeTHF**	XPhos	CyBippyPhos	PhMPhos	cataCXium A	RuPhos	QPhos	tBu3P	TFP	FcP(tBu)2	Cy3P	DPPF	DtBPF
**Toluene**	CYTOP	SPhos	ToMeOP	cataCXium PICy	FcP(tBu)2	DPPF	TXPhos	AmPhos	cataCXium A	Ad3P	XantPhos	TPP

The MTP was transferred into a nitrogen-purged glovebox and stirring bars were loaded into each vial. Using a multi-dispenser pipette, solvent (23 µL) was added to each vial, followed by an aqueous solution of K_2CO_3 (10 µL, 635 mg/mL, 46 µmol, 3.0 equiv.) or DIPEA (8 µL, 46 µmol, 3.0 equiv.) and H_2O (7.5 µL). The MTP was sealed and stirred at room temperature, 250 rpm for 16 h. The MTP was unsealed and 50 µL of a 4,4'-di-tert-butylbiphenyl internal standard solution in THF (~0.6 mg / vial) was added to each reaction mixture. The MTP was sealed, homogenized by inverting ten times to facilitate stirring, stirred for 5 min and opened. Aliquots (50 µL) of each reaction mixture were transferred to an analytical 96-well plate containing MeCN (1.0 mL / well). The resulting solutions were analyzed by HPLC using Method 1.

c. Secondary screening for the formation of 3

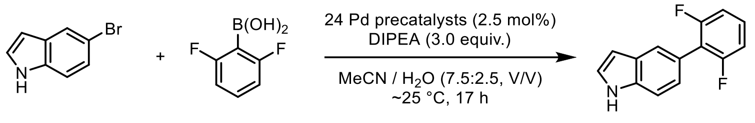

Into a Paradox 96-well microtiter plate (MTP) filled with reaction vials, stirring bars were loaded followed by Pd precatalysts on ChemBeads (10 µmol/g, 2.5 mol%).

*Table S6: Plate design (secondary screening) for the coupling between **1** and **2**. For the Pd precatalysts, only the corresponding ligands are displayed.*

	DIPEA					
**MeCN**	TPP	DPPF	DtBPF	QPhos	AmPhos	tBu3P
	XPhos	RuPhos	SPhos	XantPhos	Cy3P	cataCXium A
	cataCXium PICy	CYTOP	cataCXium POMetB	P(oTol)3	TFP	Ad3P
	PhMPhos	CycBRIDP	CyBippyPhos	ToMeOP	FcP(tBu)2	TXPhos

The MTP was transferred into a nitrogen-purged glovebox. 5-bromoindole (10 mg, 51 µmol, 1.0 equiv.) and 2,6-difluorophenylboronic acid (9.9 mg, 61 µmol, 1.2 equiv.) were dispensed to each vial as stock solutions in MeCN (75 µL), followed by DIPEA (26 µL, 153 µmol, 3.0 equiv.) and H_2O (25 µL). The MTP was sealed and stirred at ambient temperature, 250 rpm for 17 h. The MTP was unsealed and 350 µL of a 4,4'-di-tert-butylbiphenyl internal standard solution in THF (~0.4 mg / vial) was added to each reaction mixture. The MTP was sealed, homogenized by inverting ten times to facilitate stirring, stirred for 5 min and opened. Aliquots (50 µL) of each reaction mixture were transferred to an analytical 96-well plate containing MeCN (1.0 mL / well). The resulting solutions were analyzed by HPLC using Method 1.

d. Screening of Pd sources for the formation of 3

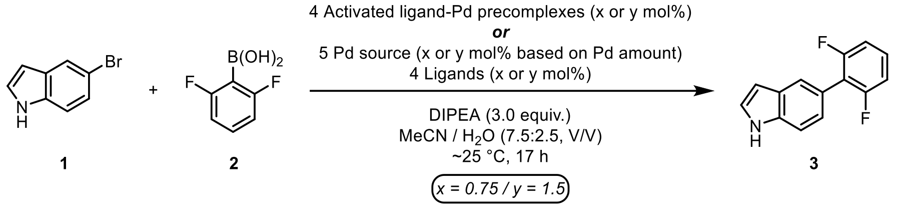

Into a Paradox 96-well microtiter plate (MTP) filled with reaction vials, stirring bars were loaded followed by Pd precatalysts on ChemBeads (10 µmol/g), Pd source on ChemBeads (20 µmol/g) and phosphines on ChemBeads (10 µmol/g) (see Table S7). The MTP was transferred into a nitrogen-purged glovebox. 5-bromoindole (10 mg, 51 µmol, 1.0 equiv.) and 2,6-difluorophenylboronic acid (9.9 mg, 61 µmol, 1.2 equiv.) were dispensed to each vial as stock solutions in MeCN (75 µL), followed by DIPEA (26 µL, 153 µmol, 3.0 equiv.) and H_2O (25 µL). The MTP was sealed and stirred at room temperature, 250 rpm for 17 h. The MTP was unsealed and 350 µL of a 4,4'-di-tert-butylbiphenyl internal standard solution in THF (~0.4 mg / vial) was added to each reaction mixture. The MTP was sealed, homogenized by inverting ten times to facilitate stirring, stirred for 5 min and opened. Aliquots (50 µL) of each reaction mixture were transferred to an analytical 96-well plate containing MeCN (1.0 mL / well). The resulting solutions were analyzed by HPLC using Method 1.

Table S7: Evaluation of Pd sources with the most active ligands identified in the primary and secondary screenings (through their corresponding Pd precomplexes) at two different Pd loadings.

Entry	Pd precatalyst	Pd precatalyst loading (equiv.)	Ligand	Ligand loading (equiv.)	Pd loading
1	P011_Pd05 | tBu3P	0.015	-	-	1.5% Pd
2	Pd02 | Pd(OAc)2	0.015	P011 | tBu3P	0.015	
3	Pd03 | Pd2(dba)3	0.0075		0.015	
4	Pd06 | [Pd(Cinnamyl)Cl]2	0.0075		0.015	
5	Pd07 | [Pd(tBu-Ind)Cl]2	0.0075		0.015	
6	Pd14 | [Pd(α-MeNAP)Br]2	0.0075		0.015	
7	P026_Pd08 | cataCXium POMetB	0.015	-	-	
8	Pd02 | Pd(OAc)2	0.015	P026 | cataCXium POMetB	0.015	
9	Pd03 | Pd2(dba)3	0.0075		0.015	
10	Pd06 | [Pd(Cinnamyl)Cl]2	0.0075		0.015	
11	Pd07 | [Pd(tBu-Ind)Cl]2	0.0075		0.015	
12	Pd14 | [Pd(α-MeNAP)Br]2	0.0075		0.015	
13	P048_Pd08 | Ad3P	0.015	-	-	
14	Pd02 | Pd(OAc)2	0.015	P048 | Ad3P	0.015	
15	Pd03 | Pd2(dba)3	0.0075		0.015	
16	Pd06 | [Pd(Cinnamyl)Cl]2	0.0075		0.015	
17	Pd07 | [Pd(tBu-Ind)Cl]2	0.0075		0.015	
18	Pd14 | [Pd(α-MeNAP)Br]2	0.0075		0.015	
19	P303+Pd06 | FcP(tBu)2	0.015	-	-	
20	Pd02 | Pd(OAc)2	0.015	P303 | tbPf	0.015	
21	Pd03 | Pd2(dba)3	0.0075		0.015	
22	Pd06 | [Pd(Cinnamyl)Cl]2	0.0075		0.015	
23	Pd07 | [Pd(tBu-Ind)Cl]2	0.0075		0.015	
24	Pd14 | [Pd(α-MeNAP)Br]2	0.0075		0.015	
25	P011_Pd05 | tBu3P	0.0075	-	-	0.75% Pd
26	Pd02 | Pd(OAc)2	0.0075	P011 | tBu3P	0.0075	
27	Pd03 | Pd2(dba)3	0.00375		0.0075	
28	Pd06 | [Pd(Cinnamyl)Cl]2	0.00375		0.0075	
29	Pd07 | [Pd(tBu-Ind)Cl]2	0.00375		0.0075	
30	Pd14 | [Pd(α-MeNAP)Br]2	0.00375		0.0075	
31	P026_Pd08 | cataCXium POMetB	0.0075	-	-	
32	Pd02 | Pd(OAc)2	0.0075	P026 | cataCXium POMetB	0.0075	
33	Pd03 | Pd2(dba)3	0.00375		0.0075	
34	Pd06 | [Pd(Cinnamyl)Cl]2	0.00375		0.0075	
35	Pd07 | [Pd(tBu-Ind)Cl]2	0.00375		0.0075	
36	Pd14 | [Pd(α-MeNAP)Br]2	0.00375		0.0075	
37	P048_Pd08 | Ad3P	0.0075	-	-	
38	Pd02 | Pd(OAc)2	0.0075	P048 | Ad3P	0.0075	
39	Pd03 | Pd2(dba)3	0.00375		0.0075	
40	Pd06 | [Pd(Cinnamyl)Cl]2	0.00375		0.0075	
41	Pd07 | [Pd(tBu-Ind)Cl]2	0.00375		0.0075	
42	Pd14 | [Pd(α-MeNAP)Br]2	0.00375		0.0075	
43	P303+Pd06 | FcP(tBu)2	0.0075	-	-	
44	Pd02 | Pd(OAc)2	0.0075	P303 | tbPf	0.0075	
45	Pd03 | Pd2(dba)3	0.00375		0.0075	
46	Pd06 | [Pd(Cinnamyl)Cl]2	0.00375		0.0075	
47	Pd07 | [Pd(tBu-Ind)Cl]2	0.00375		0.0075	
48	Pd14 | [Pd(α-MeNAP)Br]2	0.00375		0.0075	

e. Frugal sampling for the formation of 6

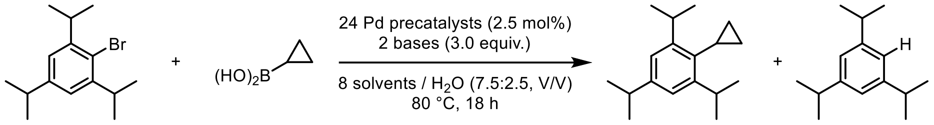

4 **5** **6** **7**

Into a Paradox 96-well microtiter plate (MTP) filled with reaction vials, stirring bars were loaded followed by Pd precatalysts on ChemBeads (10 µmol/g, 0.8 µmol, 2.5 mol%) and K_2CO_3 (14 mg, 0.10 mmol, 3.0 equiv.).

*Table S8: Plate design (primary screening) for the coupling between **4** and **5**. For the Pd precatalysts, only the corresponding ligands are displayed.*

	K2CO3						DIPEA					
**EtOH**	tBu3P	cataCXium A	XPhos	PhMPhos	DPPF	TXPhos	TPP	RuPhos	Cy3P	ToMeOP	P(oTol)3	AmPhos
**iPrOH**	FcP(tBu)2	RuPhos	P(oTol)3	TFP	XantPhos	cataCXium POMetB	PhMPhos	CYTOP	tBu3P	CyBippyPhos	XPhos	cataCXium A
**tAmOH**	QPhos	TPP	DtBPF	ToMeOP	AmPhos	CYTOP	TXPhos	cataCXium PICy	Ad3P	SPhos	CycBRIDP	DPPF
**ACN**	Cy3P	cataCXium PICy	CyBippyPhos	SPhos	CycBRIDP	Ad3P	XantPhos	TFP	QPhos	cataCXium POMetB	FcP(tBu)2	DtBPF
**NMP**	DtBPF	cataCXium POMetB	TFP	AmPhos	P(oTol)3	CycBRIDP	cataCXium PICy	RuPhos	SPhos	ToMeOP	PhMPhos	CyBippyPhos
**Dioxane**	Cy3P	tBu3P	XantPhos	TXPhos	Ad3P	TPP	P(oTol)3	CycBRIDP	QPhos	XPhos	cataCXium POMetB	CYTOP
**MeTHF**	XPhos	CyBippyPhos	PhMPhos	cataCXium A	RuPhos	QPhos	tBu3P	TFP	FcP(tBu)2	Cy3P	DPPF	DtBPF
**Toluene**	CYTOP	SPhos	ToMeOP	cataCXium PICy	FcP(tBu)2	DPPF	TXPhos	AmPhos	cataCXium A	Ad3P	XantPhos	TPP

The MTP was transferred into a nitrogen-purged glovebox. 2-bromo-1,3,5-triisopropyl-benzene (8.5 µL, 34 µmol, 1.0 equiv.) was added to each vial. Cyclopropylboronic acid (3.5 mg, 41 µmol, 1.2 equiv.) was dispensed to each vial as a suspension in H_2O (30 µL). DIPEA (17 µL, 102 µmol, 3.0 equiv.) and solvents (75 µL) were added to the appropriate vials. The MTP was sealed and stirred at 80 °C, 250 rpm for 18 h. The MTP was unsealed and 350 µL of a biphenyl internal standard solution in MeCN (~2.0 mg / vial) was added to each reaction mixture. The MTP was sealed, homogenized by inverting ten times to facilitate stirring, stirred for 5 min and opened. Aliquots (50 µL) of each reaction mixture were transferred to an analytical 96-well plate containing MeCN (1.0 mL / well). The resulting solutions were analyzed by HPLC using Method 1.

f. Secondary screening for the formation of 6

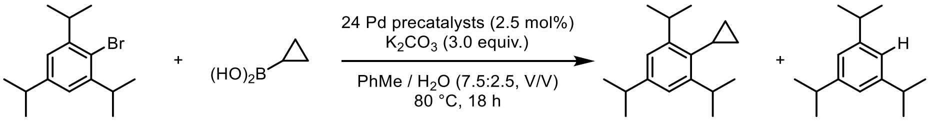

4 **5** **6** **7**

Into a Paradox 96-well microtiter plate (MTP) filled with reaction vials, stirring bars were loaded followed by Pd precatalysts on ChemBeads (10 µmol/g, 2.5 mol%) and K_2CO_3 (15 mg, 106 µmol, 3.0 equiv.).

*Table S9: Plate design (secondary screening) for the coupling between **4** and **5**. For the Pd precatalysts, only the corresponding ligands are displayed.*

	**K2CO3**					
**Toluene**	TPP	DPPF	DtBPF	QPhos	AmPhos	tBu3P
	XPhos	RuPhos	SPhos	XantPhos	Cy3P	cataCXium A
	cataCXium PlCy	CYTOP	cataCXium POMetB	P(oTol)3	TFP	Ad3P
	PhMPhos	CycBRIDP	CyBippyPhos	ToMeOP	FcP(tBu)2	TXPhos

The MTP was transferred into a nitrogen-purged glovebox. 2-bromo-1,3,5-triisopropyl-benzene (10 mg, 35 µmol, 1.0 equiv.) was added to each vial. Cyclopropylboronic acid (3.6 mg, 42 µmol, 1.2 equiv.) was dispensed to each vial as a suspension in H_2O (30 µL). Toluene (75 µL) was added to the appropriate vials. The MTP was sealed and stirred at 80 °C, 250 rpm for 18 h. The MTP was unsealed and 350 µL of a biphenyl internal standard solution in MeCN (~2.0 mg / vial) was added to each reaction mixture. The MTP was sealed, homogenized by inverting ten times to facilitate stirring, stirred for 5 min and opened. Aliquots (50 µL) of each reaction mixture were transferred to an analytical 96-well plate containing MeCN (1.0 mL / well). The resulting solutions were analyzed by HPLC using Method 1.

g. DoE for the formation of 6

The effect of some continuous reaction parameters was evaluated in a full factorial DoE at reduced catalyst (1.5 mol%) loading, using the practical $PdCl_2(PPh_3)_2$ (**P001_Pd001**), which provided the highest conversion in the previous screening.

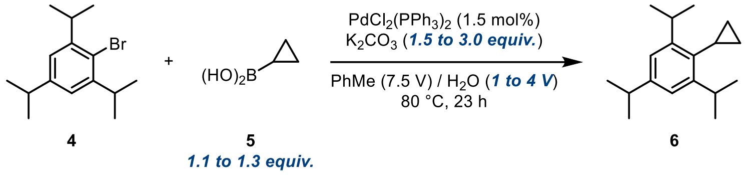

Into HPLC vials, stirring bars were loaded followed by $PdCl_2(PPh_3)_2$ (3 mg, 5.3 µmol, 1.5 mol%), K_2CO_3 (73 – 46 mg, 0.53 – 1.06 mmol, 1.5 – 3.0 equiv.) and cyclopropylboronic acid (33 – 39 mg, 0.39 – 0.46 mmol, 1.1 – 1.3 equiv.). The MTP was transferred into a nitrogen-purged glovebox. 2-bromo-1,3,5-triisopropyl-benzene (100 mg, 0.35 mmol, 1.0 equiv.) and 750 µL of a biphenyl internal standard solution in toluene (~3.0 mg / vial) were added to each vial. H_2O (100 – 400 µL, 1 – 4 V) was added to the appropriate vials. The reaction mixtures were sealed and stirred at 80 °C, 250 rpm for 23 h. After allowing the reaction mixtures to return to ambient temperature, they were transferred to larger vials and diluted with MeCN (3 mL). Aliquots (50 µL) of each reaction mixture were diluted with MeCN (1.0 mL). The resulting solutions were analyzed by HPLC using Method 1.

Table S10 : Factor settings and measured responses as calculated assay yields for the full factorial design.

Std	Run	Space	Factor A RB(OH)2 **5** (mol%)	Factor B K_2CO_3 (mol%)	Factor C H_2O (V)	Yield of ArBr **4** (%)	Yield of Prod **6** (%)
1	8	Factorial	110	150	1,0	22	61
2	1	Factorial	130	150	1,0	17	65
3	4	Factorial	110	300	1,0	0	81
4	9	Factorial	130	300	1,0	0	79
5	2	Factorial	110	150	4,0	65	27
6	6	Factorial	130	150	4,0	59	26
7	11	Factorial	110	300	4,0	43	53
8	10	Factorial	130	300	4,0	47	47
9	12	Center	120	225	2,5	26	62
10	3	Center	120	225	2,5	24	65
11	7	Center	120	225	2,5	37	56
12	5	Center	120	225	2,5	30	60

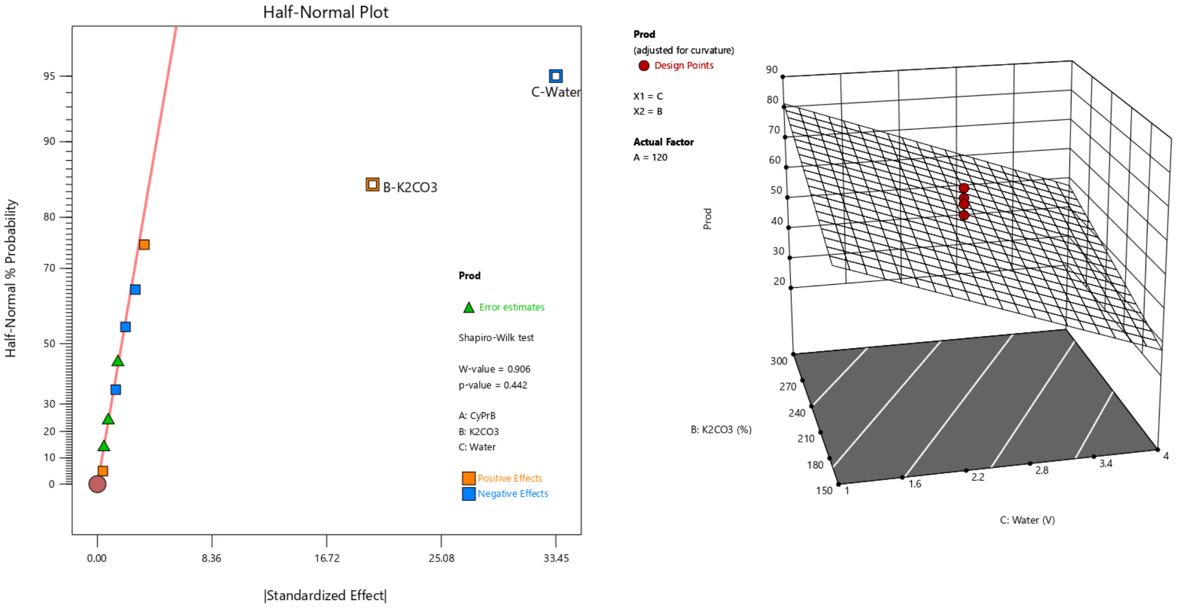

Figure S2: Half-normal plot (left) and response surface (right) for full factorial design showing the negative effect of H_2O and positive effect of K_2CO_3 on the calculated product assay yields.

Table S11: ANOVA table for the selected factorial design, which indicates a significant model, with B and C as significant model terms, as well as a statistically significant curvature (p value <0.05).

Source	Sum of Squares	df	Mean Square	F-value	p-value	
**Model**	3043,69	2	1521,8	137,385	<0.0001	significant
B-K_2CO_3	806,46	1	806,5	72,804	<0.0001	
C-H_2O	2237,23	1	2237,2	201,966	<0.0001	
Curvature	100,05	1	100,0	9,032	0,0169	
**Residual**	88,62	8	11,1			
Lack of Fit	51,01	5	10,2	0,814	0,6090	not significant
Pure Error	37,60	3	12,5			
**Cor Total**	3232,36	11				

Table S12: Fit statistics, which confirm that a predictive model can be used to navigate the design space.

**Std. Dev.**	33,3	**R^2**	0,9717
**Mean**	57	**Adjusted R^2**	0,9646
**C.V. %**	6	**Predicted R^2**	0,9370
		**Adeq Precision**	27,8556

4. Buchwald-Hartwig – coupling between 8 and 9

a. Pd precatalysts

*Table S13: Pd precatalysts employed for the coupling between **8** and **9**.*

Entry	Internal Acronym	CAS N°	MW (g/mol)	Name
1	P006_Pd01 | DIPPF	215788-65-1	595.64	1,1′-Bis(di-isopropylphosphino)ferrocene palladium dichloride
2	P009_Pd05 | QPhos	1252598-33-6	907.69	Chloro(crotyl)[1,2,3,4,5-pentaphenyl-1'-(di-tert-butylphosphino)ferrocene]palladium(II)
3	P010_Pd05 | AmPhos	1334497-06-1	462.35	Chloro(crotyl)[di-tert-butyl(4-dimethylaminophenyl)phosphine]palladium(II)
4	P012_Pd05 | XPhos	1798782-02-1	673.70	Chloro(crotyl)(2-dicyclohexylphosphino-2',4',6'-triisopropyl-1,1'-biphenyl) palladium(II)
5	P013_Pd05 | RuPhos	1798781-96-0	663.62	Pd(crotyl)Cl;Chloro(crotyl)[2-Dicyclohexylphosphino-2',6'-di-i-sopropoxy-1,1'- biphenyl]palladium(II)
6	P014_Pd05 | SPhos	1798781-99-3	607.51	[(1,2,3-η)-2-Buten-1-yl]chloro[dicyclohexyl(2',6'-dimethoxy[1,1'-biphenyl]-2-yl)phosphine-κP]palladium
7	P015_Pd05 | BrettPhos	1798782-11-2	847.36	[(1,2,3-η)-2-Buten-1-yl][dicyclohexyl[3,6-dimethoxy-2',4',6'-tris(1-methylethyl)[1,1'-biphenyl]-2-yl-κC1']phosphine-κP]-Palladium(1+), 1,1,1-trifluoromethanesulfonate
8	P016_Pd04 | tBuXPhos	1798782-25-8	721.21	[Bis(1,1-dimethylethyl)[2′,4′,6′-tris(1-methylethyl)[1,1′-biphenyl]-2-yl-κC1′]phosphine-κP][(1,2,3-η)-2-buten-1-yl]-Palladium(1+) 1,1,1-trifluoromethanesulfonate
9	P017_Pd05 | tBuBrettPhos	1798782-15-6	781.26	[[3,6-Dimethoxy-2',4',6'-tris(1-methylethyl)[1,1'-biphenyl]-2-yl-κC1']bis(1,1-dimethylethyl)phosphine-κP](η3-2-propen-1-yl)-Palladium(1+), 1,1,1-trifluoromethanesulfonate
10	P018_Pd04 | BINAP	879689-47-1	805.63	[(1R)-[1,1'-Binaphthalene]-2,2'-diylbis[diphenylphosphine-κP]](η3-2-propenyl)-Palladium chloride
11	P019_Pd04 | XantPhos	879689-28-8	761.57	Palladium, chloro[(9,9-dimethyl-9H-xanthene-4,5-diyl)bis[diphenylphosphine-κP]](η3-2-propenyl)-
12	P020_Pd05 | Cy3P	307494-95-7	477.41	[(1,2,3-η)-2-Butenyl]chloro(tricyclohexylphosphine)palladium
13	P021_Pd04 | BippyPhos	1846558-62-0	803.19	[5-[Bis(1,1-dimethylethyl)phosphino-κP]-1',3',5'-triphenyl-1,4'-bi-1H-pyrazole][(1,2,3-η)-1-phenyl-2-propen-1-yl]-Palladium(1+), 1,1,1-trifluoromethanesulfonate
14	P022_Pd04 | cataCXium A	2703751-81-7	541.49	Pd(Ad_2P(n-Bu))(allyl)Cl
15	P023_Pd04 | JosiPhos	n.a.	851.12	Pd(Josiphos SL-J009-1)(allyl)Cl
16	P024_PdG3 | CYTOP	1350851-22-7	602.40	Chloro[(1,3,5,7-tetramethyl-5-phenyl-2,4,8-trioxa-6-phosphaadamantane)-2-(2-aminobiphenyl)]palladium(II)
17	P025_Pd05 | cataCXium PICy	n.a.	692.12	cataCXium PICy Pd(crotyl)OTf
18	P026_Pd08 | cataCXium POMetB	n.a.	730.18	(2-acetamidophenyl)(di-tert-butyl(1-(2-methoxyphenyl)-1H-pyrrol-2-yl)-l5-phosphaneyl)palladium, 4-methylbenzenesulfonate salt
19	P028_Pd05 | cataCXium PtB	n.a.	484.11	cataCXium PtB Pd(crotyl)Cl
20	P032_Pd01 | DPPB	29964-62-3	603.80	1,4-Butylenebis(diphenylphosphine)palladium dichloride
21	P033_Pd01 | DCyPP	1041005-52-0	613.96	Dichloro[bis(dicyclohexylphosphino)propane]palladium(II)
22	P036_Pd04 | Me4tBuXphos	n.a.	777.31	Me_4tBuXphos Pd(allyl)OTf
23	P038_PdG3 | DavePhos	1445085-87-9	763.28	Methanesulfonato[2-(dicyclohexylphosphino)-2'-(N,N-dimethylamino)-1,1'-biphenyl](2'-amino-1,1'-biphenyl-2-yl)palladium(II)
24	P043_Pd04 | AlPhos	2097600-18-3	1111.63	AlPhos Pd(allyl)OTf

b. Frugal sampling for the formation of 10

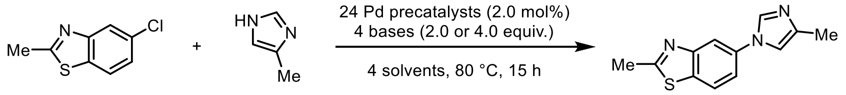

5-chloro-2-methyl-1,3-benzothiazole (3.5 mg, 19 µmol, 1.0 equiv.) and 4-methylimidazole (1.9 mg, 23 µmol, 1.2 equiv.) were dispensed as stock solutions in CH_2Cl_2 into a Paradox 96-well microtiter plate (MTP) filled with reaction vials. The solvent was removed under a nitrogen stream. Pd precatalysts on ChemBeads (10 µmol/g, 2.0 mol%) were added sequentially to the appropriate vials:

*Table S14: Plate design (primary screening) for the coupling between **8** and **9**. For the Pd precatalysts, only the corresponding ligands are displayed.*

	K3PO4			Cs2CO3			NaOTMS			NaOtAm		
tAmOH	DPPB	tBuXPhos	SPhos	tBuBrettPhos	RuPhos	AlPhos	cataCXium PlCy	JosiPhos	Me4tBuXphos	XantPhos	cataCXium PtB	QPhos
	BrettPhos	cataCXium POMetB	BippyPhos	Cy3P	cataCXium A	CYTOP	DavePhos	DIPPF	DCyPP	AmPhos	XPhos	BINAP
MeTHF	XantPhos	Me4tBuXphos	BINAP	JosiPhos	DPPB	tBuXPhos	BippyPhos	AmPhos	QPhos	DIPPF	Cy3P	cataCXium A
	AlPhos	cataCXium PlCy	CYTOP	XPhos	SPhos	cataCXium PtB	RuPhos	cataCXium POMetB	tBuBrettPhos	DCyPP	DavePhos	BrettPhos
NMP	DCyPP	cataCXium PtB	AmPhos	DavePhos	cataCXium PlCy	Me4tBuXphos	tBuXPhos	XantPhos	cataCXium A	BippyPhos	JosiPhos	CYTOP
	XPhos	QPhos	Cy3P	DIPPF	cataCXium POMetB	BrettPhos	DPPB	BINAP	SPhos	AlPhos	RuPhos	tBuBrettPhos
Toluene	cataCXium A	RuPhos	DavePhos	XantPhos	QPhos	AmPhos	AlPhos	CYTOP	Cy3P	cataCXium PlCy	cataCXium POMetB	DPPB
	DIPPF	tBuBrettPhos	JosiPhos	BippyPhos	BINAP	DCyPP	cataCXium PtB	XPhos	BrettPhos	Me4tBuXphos	SPhos	tBuXPhos

K_3PO_4 (16 mg, 76 µmol, 4.0 equiv.) and Cs_2CO_3 (25 mg, 76 µmol, 4.0 equiv.) were added to the appropriate vials. The MTP was transferred into a nitrogen-purged glovebox and stirring bars were loaded into each vial. Using a multi-dispenser pipette, solvent (100 µL) was added to the appropriate vials, followed by NaOTMS (38 µL, 1 M, 38 µmol, 2.0 equiv.) and NaOtAm (10 µL, 40 %, 38 µmol, 2.0 equiv.). The MTP was sealed and stirred at 80 °C, 250 rpm for 15 h. The MTP was unsealed and 350 µL of a biphenyl internal standard solution in MeCN (~2.3 mg / vial) was added to each reaction mixture. The MTP was sealed, homogenized by inverting ten times to facilitate stirring, stirred for 5 min and opened. Aliquots (50 µL) of each reaction mixture were transferred to an analytical 96-well plate containing MeCN (1.0 mL / well). The resulting solutions were analyzed by HPLC using Method 1.

5. Buchwald-Hartwig – coupling between 11 and 12

a. Pd precatalysts

*Table S15: Pd precatalysts employed for the coupling between **11** and **12**. [a] Physical mixture of [Pd(cinnamyl)Cl]$_2$ and the ligand; CAS N° of the ligand and MW of the mixture are displayed.*

Entry	Internal Acronym	Ligand of interest	CAS N°	MW (g/mol)	Name
1	P003_Pd01	dppf	138549-82-3	760.74	[1,1'-Bis(diphenylphosphino)ferrocene]dichloropalladium(II)
2	P004_Pd01	DPEPhos	205319-06-8	715.88	Dichloro[bis(2-(diphenylphosphino)phenyl)ether]palladium(II)
3	P005_Pd01	dtbpf	95408-45-0	651.75	1,1'-Bis-(di-tert.-butylphosphino-)ferrocen-palladiumdichloride
4	P006_Pd01	dippf	215788-65-1	595.64	1,1'-Bis(di-isopropylphosphino)ferrocene palladium dichloride
5	P007_Pd01	dppp	59831-02-6	589.77	1,3-Bis(diphenylphosphino)propane)palladium(II)chloride
6	P009_Pd05	QPhos	1252598-33-6	907.69	Chloro(crotyl)[1,2,3,4,5-pentaphenyl-1'-(di-tert-butylphosphino)ferrocene]palladium(II)
7	P010_Pd05	AmPhos	1334497-06-1	462.35	Chloro(crotyl)[di-tert-butyl(4-dimethylaminophenyl)phosphine]palladium(II)
8	P012_Pd05	XPhos	1798782-02-1	673.70	Chloro(crotyl)(2-dicyclohexylphosphino-2',4',6'-triisopropyl-1,1'-biphenyl) palladium(II)
9	P013_Pd05	RuPhos	1798781-96-0	663.62	Pd(crotyl)Cl;Chloro(crotyl)[2-Dicyclohexylphosphino-2',6'-di-i-sopropoxy-1,1'- biphenyl]palladium(II)
10	P014_Pd05	SPhos	1798781-99-3	607.51	[(1,2,3-η)-2-Buten-1-yl]chloro[dicyclohexyl(2',6'-dimethoxy[1,1'-biphenyl]-2-yl)phosphine-κP]palladium
11	P015_Pd05	BrettPhos	1798782-11-2	847.36	[(1,2,3-η)-2-Buten-1-yl][dicyclohexyl[3,6-dimethoxy-2',4',6'-tris(1-methylethyl)[1,1'-biphenyl]-2-yl-κC1']phosphine-κP]-Palladium(1+), 1,1,1-trifluoromethanesulfonate
12	P017_Pd05	tBuBrettPhos	1798782-15-6	781.26	[[3,6-Dimethoxy-2',4',6'-tris(1-methylethyl)[1,1'-biphenyl]-2-yl-κC1']bis(1,1-dimethylethyl)phosphine-κP](η3-2-propen-1-yl)-Palladium(1+), 1,1,1-trifluoromethanesulfonate
13	P018_Pd04	BINAP	879689-47-1	805.63	[(1R)-[1,1'-Binaphthalene]-2,2'-diylbis[diphenylphosphine-κP]](η3-2-propenyl)-Palladium chloride
14	P019_Pd04	XantPhos	879689-28-8	761.57	Palladium, chloro[(9,9-dimethyl-9H-xanthene-4,5-diyl)bis[diphenylphosphine-κP]](η3-2-propenyl)-
15	P021_Pd04	BippyPhos	1846558-62-0	803.19	[5-[Bis(1,1-dimethylethyl)phosphino-κP]-1',3',5'-triphenyl-1,4'-bi-1H-pyrazole][(1,2,3-η)-1-phenyl-2-propen-1-yl]-Palladium(1+), 1,1,1-trifluoromethanesulfonate
16	P022_Pd04	cataCXium A	2703751-81-7	541.49	Pd(Ad$_2$P(n-Bu))(allyl)Cl
17	P023_Pd04	JosiPhos SL-J009-1	n.a.	851.12	Pd(Josiphos SL-J009-1)(allyl)Cl
18	P025_Pd05	cataCXium PICy	n.a.	692.12	cataCXium PlCy Pd(crotyl)OTf
19	P026_Pd08	cataCXium POMetB	n.a.	730.18	(2-acetamidophenyl)(di-tert-butyl(1-(2-methoxyphenyl)-1H-pyrrol-2-yl)-l5-phosphaneyl)palladium, 4-methylbenzenesulfonate salt
20	P030_Pd04	AdBippyPhos	n.a.	959.42	AdBippyPhos Pd(all)OTf
21	P031_Pd01	dppe	19978-61-1	575.75	[1,2-Bis(diphenylphosphino)ethane]dichloropalladium(II)
22	P032_Pd01	dppb	29964-62-3	603.80	1,4-Butylenebis(diphenylphosphine)palladium dichloride
23	P036_Pd04	Me4tBuXPhos	n.a.	777.31	Me4tBuXphos Pd(allyl)OTf

24	P039_Pd04	RockPhos	1798782-31-6	765.26	[Bis(1,1-dimethylethyl)[3-methoxy-6-methyl-2',4',6'-tris(1-methylethyl)[1,1'-biphenyl]-2-yl-κC1']phosphine-κP](η3-2-propen-1-yl)palladium 1,1,1-trifluoromethanesulfonate
25	NHC040_Pd09	Cl2-iPent-NHC	1612891-29-8	840.15	[1,3-BIs[2,6-bis(1-ethylpropyl)phenyl]-4,5-dichloro-1,3-dihydro-2H-imidazol-2-ylidene]dichloro(2-methylpyridine)palladium
26	P041_Pd04	JackiePhos	n.a.	1093.23	[2'-(Amino-κN)[1,1'-biphenyl]-2-yl-κC][bis[3,5-bis(trifluoromethyl)phenyl][3,6-dimethoxy-2',4',6'-tris(1-methylethyl)[1,1'-biphenyl]-2-yl]phosphine-κP](methanesulfonato-κO)palladium
27	P042_PdG3	MorDalphos	2222690-89-1	834.37	(2-(Di-1-adamantylphosphino)morpholinobenzene)[2-(2′-amino-1,1′-biphenyl)]palladium(II) methanesulfonate
28	P043_Pd04	AlPhos	2097600-18-3	1111.63	AlPhos Pd(allyl)OTf
29	P044_PdG4	tBuPhCPhos	2896826-14-3	788.29	2'-[(1,1-Dimethylethyl)phenylphosphino-κP]-N2,N2,N6,N6-tetramethyl[1,1'-biphenyl]-2,6-diamine](methanesulfonato-κO)[2'-(methylamino-κN)[1,1'-biphenyl]-2-yl-κC]Palladium
30	P047_Pd04	GPhos	n.a.	920.53	GPhos Pd(allyl)OTf
31	P048_Pd08	Ad3P	1926980-77-9	848.42	[2-[(Acetyl-κO)amino]phenyl-κC][tris(tricyclo[3.3.1.13,7]dec-1-yl)phosphine]palladium 4-methylbenzenesulfonate
32	P049_Pd04	AdBrettPhos	n.a.	937.49	AdBrettPhos Pd(allyl)OTf
33	P053+Pd03[a]	trYPhos	2271302-83-9	910,56	trYPhos+1/2 $Pd_2(dba)_3$
34	P054_PdG4	N-XantPhos	1878105-23-7	935.31	(SP-4-4)-[4-(Diphenylphosphino-κP)-6-(diphenylphosphino)-10H-phenoxazine](methanesulfonato-κO)[2'-(methylamino-κN)[1,1'-biphenyl]-2-yl-κC]palladium
35	P055_Pd04	CPhos	n.a.	733.18	CPhos Pd(allyl)OTf
36	P056_Pd04	EPhos	n.a.	831.36	EPhos Pd(allyl)OTf
37	P057_Pd04	CPhos hybrid	n.a.	1053.12	CPhos hybrid Pd(allyl)OTf
38	P058+Pd06[a]	RuPhos hybrid	1810068-31-5	1046.64	RuPhos hybrid+ 1/2$[Pd(cinnamyl)Cl]_2$
39	P061+Pd06[a]	cBRIDP	742103-27-1	611.54	cBRIDP+ 1/2$[Pd(cinnamyl)Cl]_2$
40	P062_Pd01	PhMPhos	2768152-19-6	847.95	1-Diadamantylphosphino-1′-diphenylphosphinoferrocene palladium dichloride
41	P064+Pd06[a]	CycBRIDP	1023330-38-2	663.62	CycBRIDP+ 1/2$[Pd(cinnamyl)Cl]_2$
42	P067+Pd06[a]	FPhos	3059853-80-1	777.52	FPhos+ 1/2$[Pd(cinnamyl)Cl]_2$
43	P068_Pd04	CataCXium PInCy	n.a.	686.08	cataCXium PInCy Pd(allyl)OTf
44	P069_Pd04	cataCXium POMeCy	n.a.	666.04	cataCXium POMeCy Pd(allyl)OTf
45	P070_Pd04	CyJohnPhos	n.a.	647.04	CyJohnPhos Pd(allyl)OTf
46	P071_Pd04	JohnPhos	n.a.	594.96	JohnPhos Pd(allyl)OTf
47	P072_Pd04	PhDavePhos	n.a.	678.01	PhDavePhos Pd(allyl)OTf
48	P073_Pd04	PhSPhos	n.a.	695.00	PhSPhos Pd(allyl)OTf

b. Comparison between Sobol and Latin hypercube samplings

For a perfectly balanced distribution, each ligand should be employed 4 times once in each solvent and once in each base (four different base-solvent pairs). Per solvent and base, all 48 ligands should appear exactly once.

- In both sampling methods, each ligand is employed 4 times.
- Sobol sampling:

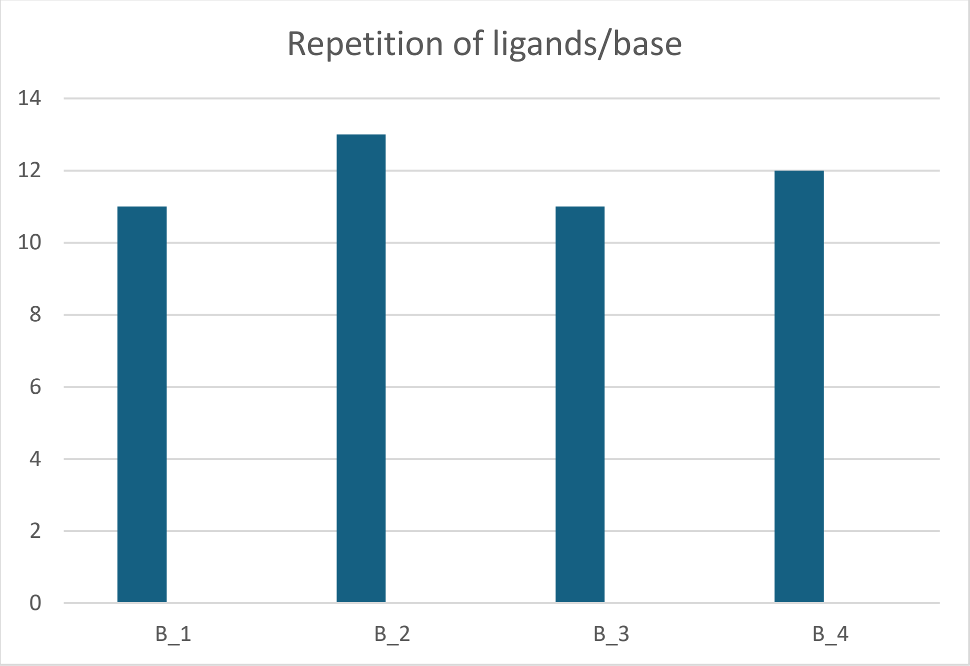

Figure S3: Number of ligands employed twice in the same base.

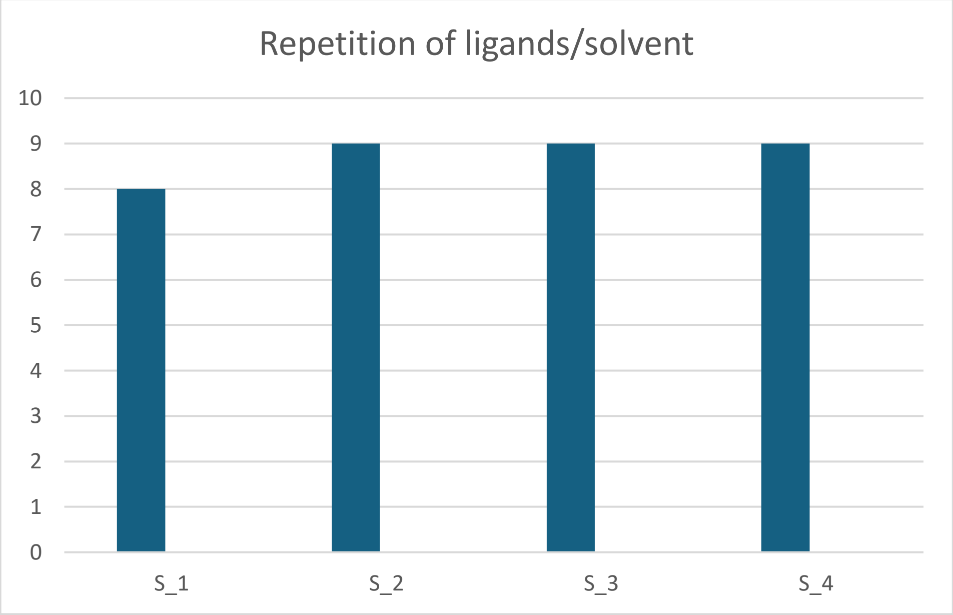

Figure S4: Number of ligands employed twice in the same solvent.

- Latin hypercube sampling:

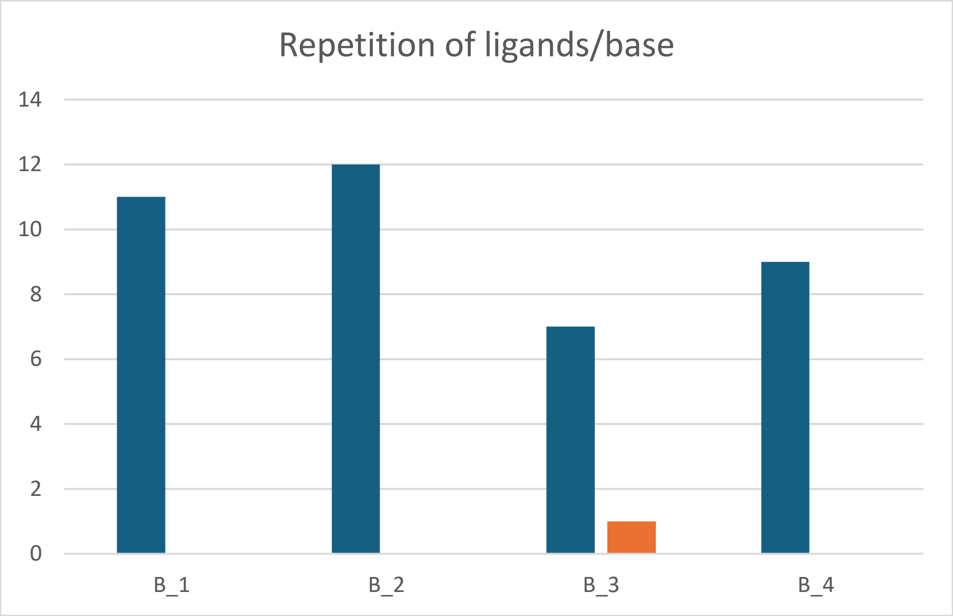

Figure S5: Number of ligands employed twice (blue) or three times (orange) in the same base.

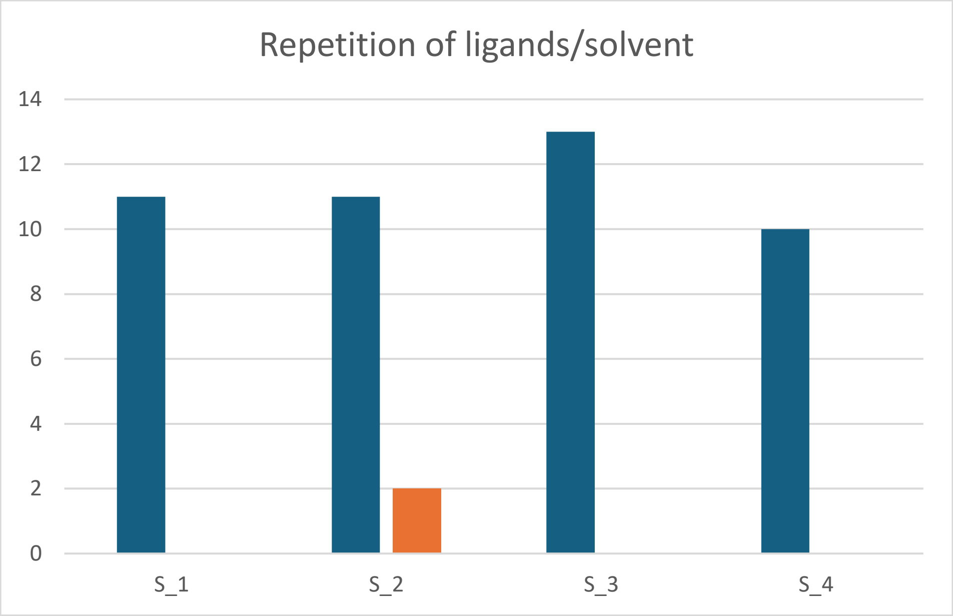

Figure S6: Number of ligands employed twice (blue) or three times (orange) in the same solvent.

c. Frugal sampling for the formation of 13

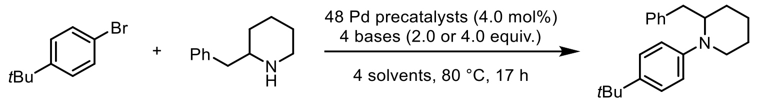

11 **12** **13**

Pd precatalysts on ChemBeads (20 mg at 10 µmol/g, 2.0 mol%), K_3PO_4 (4.0 mg, 19 µmol, 4.0 equiv.) and Cs_2CO_3 (6.1 mg, 19 µmol, 4.0 equiv.) were added sequentially to the appropriate vials of a Paradox 96-well microtiter plate (MTP).

*Table S16: Plate design (primary screening) for the coupling between **11** and **12**. For the Pd precatalysts, only the ligands of interest are displayed.*

Plate 1		Cs2CO3						K3PO4					
		**1**	**2**	**3**	**4**	**5**	**6**	**7**	**8**	**9**	**10**	**11**	**12**
**Dioxan**	A	Amphos	cataCXium PInCy	Di-Cl-PEPPSI-iPent	EPhos	JosiPhos	JohnPhos	DPPE	GPhos	CPhos	cataCXium PICy	DPPP	DPEPhos
	B	DPPB	tBuPHCPHO	F-Phos	tBuBrettPhos	CPhos	MordalPhos	RuPhos	CBRIDP	AdBrettPhos	Adamanthyl-BIPPYPHOS	QPhos	XantPhos
**Toluol**	C	AdBrettPhos	DPPF	QPhos	BINAP	N-XantPhos	AlPhos	BrettPhos	JackiePhos	Ph-SPhos	RuPhos Hybrid	BIPPYPHOS	DTBPF
	D	(PhMPhos)PdCl2	SPhos	DPPE	Ad3P	cataCXium PICy	CyJohnPhos	cataCXium POMetB	Amphos	cataCXium POMeCy	Di-Cl-PEPPSI-iPent	CPhos Hybrid	CBRIDP
**MeTHF**	E	JackiePhos	N-XantPhos	tBuBrettPhos	CBRIDP	DPPF	DiPPF	BINAP	Ph-SPhos	Adamanthyl-BIPPYPHOS	AdBrettPhos	BrettPhos	CY-cBRIDP
	F	XantPhos	Ph-DavePhos	EPhos	Di-Cl-PEPPSI-iPent	cataCXium POMeCy	XPhos	DPEPhos	trYPhos	DPPP	cataCXium PICy	Ad3P	DPPB
**tAmOH**	G	DPPP	JosiPhos	Ad3P	DPPE	cataCXium PInCy	RuPhos	EPhos	MordalPhos	cataCXium POMeCy	Amphos	GPhos	Me4tBuXPhos
	H	CPhos Hybrid	RockPhos	BINAP	QPhos	Adamanthyl-BIPPYPHOS	DTBPF	CyJohnPhos	cataCXium A	cataCXium POMetB	CPhos	tBuBrettPhos	(PhMPhos)PdCl2
**Plate 2**		NaOtAm						NaOTMS					
		**1**	**2**	**3**	**4**	**5**	**6**	**7**	**8**	**9**	**10**	**11**	**12**
**Dioxan**	A	Ph-DavePhos	XantPhos	DiPPF	cataCXium POMetB	(PhMPhos)PdCl2	tBuBrettPhos	trYPhos	AlPhos	CY-cBRIDP	SPhos	DTBPF	Adamanthyl-BIPPYPHOS
	B	N-XantPhos	cataCXium PInCy	JackiePhos	cataCXium A	JohnPhos	Me4tBuXPhos	Ph-SPhos	BINAP	RockPhos	RuPhos Hybrid	RuPhos	N-XantPhos
**Toluol**	C	RockPhos	CPhos Hybrid	XPhos	F-Phos	DPPB	Ad3P	cataCXium A	CyJohnPhos	Me4tBuXPhos	tBuPHCPHO	XPhos	cataCXium POMeCy
	D	JosiPhos	DPPP	trYPhos	AlPhos	CY-cBRIDP	BrettPhos	DPEPhos	EPhos	Ph-DavePhos	BIPPYPHOS	DiPPF	DPPF
**MeTHF**	E	tBuPHCPHO	DPPB	JohnPhos	cataCXium A	CPhos Hybrid	Di-Cl-PEPPSI-iPent	F-Phos	XPhos	RuPhos Hybrid	RockPhos	CyJohnPhos	cataCXium PICy
	F	Amphos	JackiePhos	DiPPF	BIPPYPHOS	Ph-SPhos	GPhos	GPhos	DPPE	SPhos	CY-cBRIDP	MordalPhos	cataCXium PInCy
**tAmOH**	G	SPhos	(PhMPhos)PdCl2	AlPhos	trYPhos	XantPhos	QPhos	DTBPF	cataCXium POMetB	BIPPYPHOS	Ph-DavePhos	MordalPhos	CPhos
	H	DPPF	AdBrettPhos	F-Phos	RuPhos	RuPhos Hybrid	DPEPhos	BrettPhos	CBRIDP	tBuPHCPHO	Me4tBuXPhos	JohnPhos	JosiPhos

The MTP was transferred into a nitrogen-purged glovebox and stirring bars were loaded into each vial. 1-bromo-4-tert-butyl-benzene (1.0 mg, 4.7 µmol, 1.0 equiv.) and 2-benzylpiperidine (1.1 mg, 6.1 µmol, 1.3 equiv.) were dispensed as stock solutions in the appropriate solvent (30 µL dioxane / toluene / MeTHF / *t*AmOH). NaOTMS (9.4 µL, 1 M in THF, 9.4 µmol, 2.0 equiv.) and NaO*t*Am (6.7 µL, 1.4 M in THF, 9.4 µmol, 2.0 equiv.) were added to the appropriate vials. The MTP was sealed and stirred at 80 °C, 250 rpm for 17 h. The MTP was unsealed and 80 µL of a biphenyl internal standard solution in MeCN/H_2O (8:2; ~0.2 mg / vial) was added to each reaction mixture. The MTP was sealed, homogenized by inverting ten times to facilitate stirring, stirred for 5 min and opened. Aliquots (30 µL) of each reaction mixture were transferred to an analytical 96-well plate containing MeCN/H_2O (9:1; 1.0 mL / well). The resulting solutions were analyzed by HPLC using Method 1.

d. General procedure for the LHS sampling and BO experiments – synthesis of 13

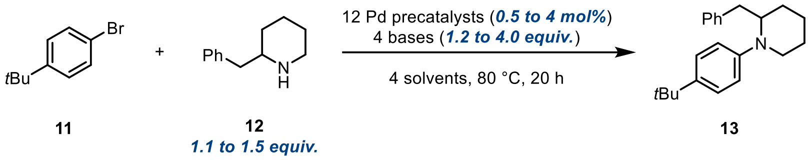

For detailed plating and results, please see additional file.

Pd precatalysts on ChemBeads (23 to 188 mg at 10 µmol/g, 0.5 – 4 mol%), K_3PO_4 (12 – 40 mg, 56 – 188 µmol, 1.2 – 4.0 equiv.) and Cs_2CO_3 (18 – 61 mg, 56 – 188 µmol, 1.2 – 4.0 equiv.) were added sequentially to the appropriate vials of a Paradox 96-well microtiter plate (MTP). The MTP was transferred into a nitrogen-purged glovebox and stirring bars were loaded into each vial. 1-bromo-4-tert-butyl-benzene (8.0 µL, 47 µmol, 1.0 equiv.) and 2-benzylpiperidine (9.2 – 12.6 µL, 5.2 – 7.0 µmol, 1.1 – 1.5 equiv.) were dispensed followed by the adequate solvents (10 – 30 µL dioxane / toluene / MeTHF / *t*AmOH). NaOTMS (56 – 188 µL, 1M in THF, 56 – 188 µmol, 1.2 – 4.0 equiv.) and NaO*t*Am (10 – 21 mg, 56 – 188 µmol, 2.0 – 4.0 equiv.) were added to the appropriate vials. The MTP was sealed and stirred at 80 °C, 250 rpm for 20 h. The MTP was unsealed and 200 µL of a biphenyl internal standard solution in MeCN (~2.0 mg / vial) was added to each reaction mixture. The MTP was sealed, homogenized by inverting ten times to facilitate stirring, stirred for 5 min and opened. Aliquots (50 µL) of each reaction mixture were transferred to an analytical 96-well plate containing MeCN/H_2O (9:1; 1.0 mL / well). The resulting solutions were analyzed by HPLC using Method 1.

e. Bayesian optimization visualization

The following plots were generated using SuntheticsML platform.

i. Using data from the frugal sampling (192 experiments)

We initially considered the frugal sampling dataset to initialize a Bayesian surrogate model. However, we anticipated that the limited diversity in continuous variable values could introduce bias into the design space exploration.

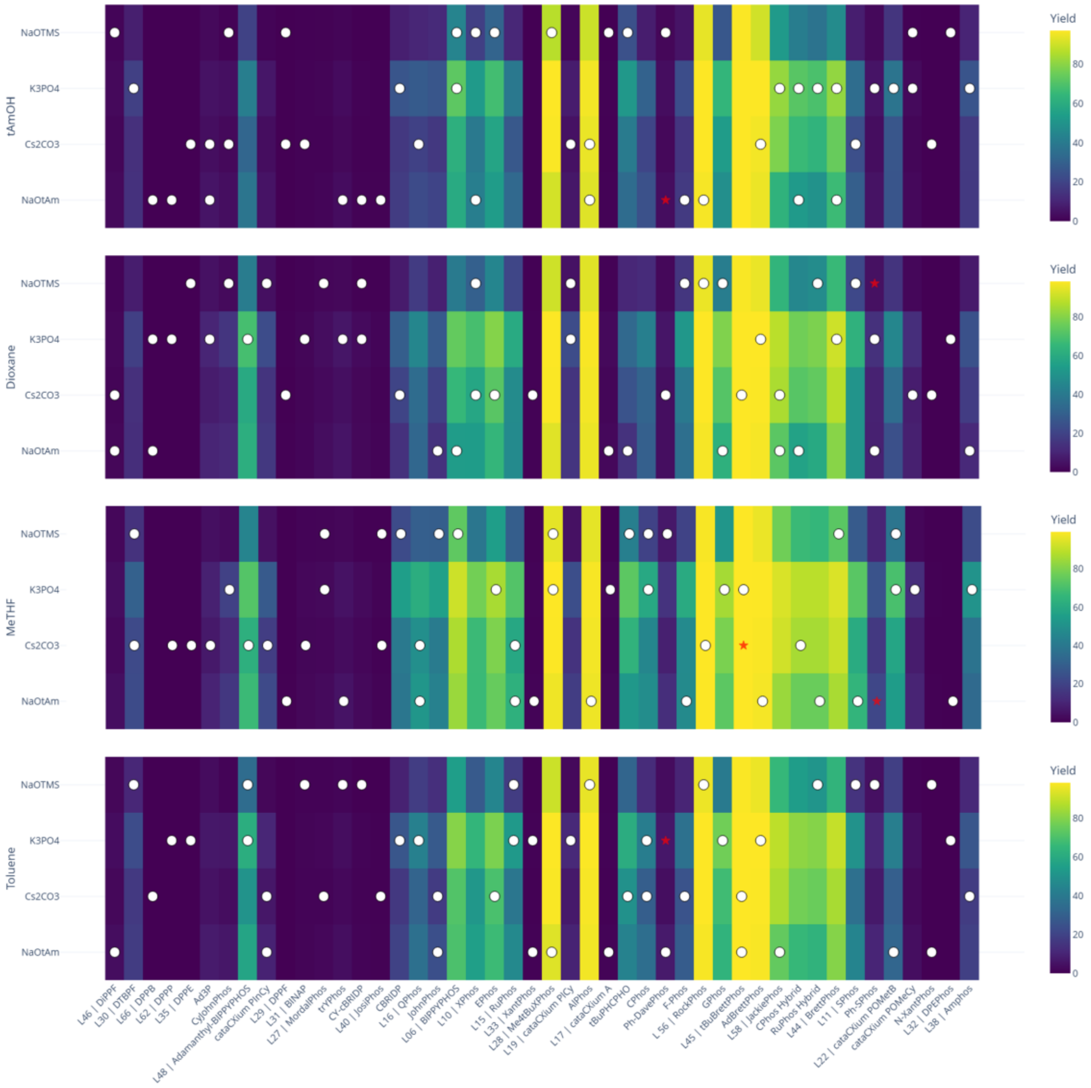

Figure S7: Data visualization after performing the frugal sampling (Sobol, 192 experiments) with solvent as the main y axis, and bases as the secondary y axis. White dots = experimental datapoints, red stars = recommended experiments.

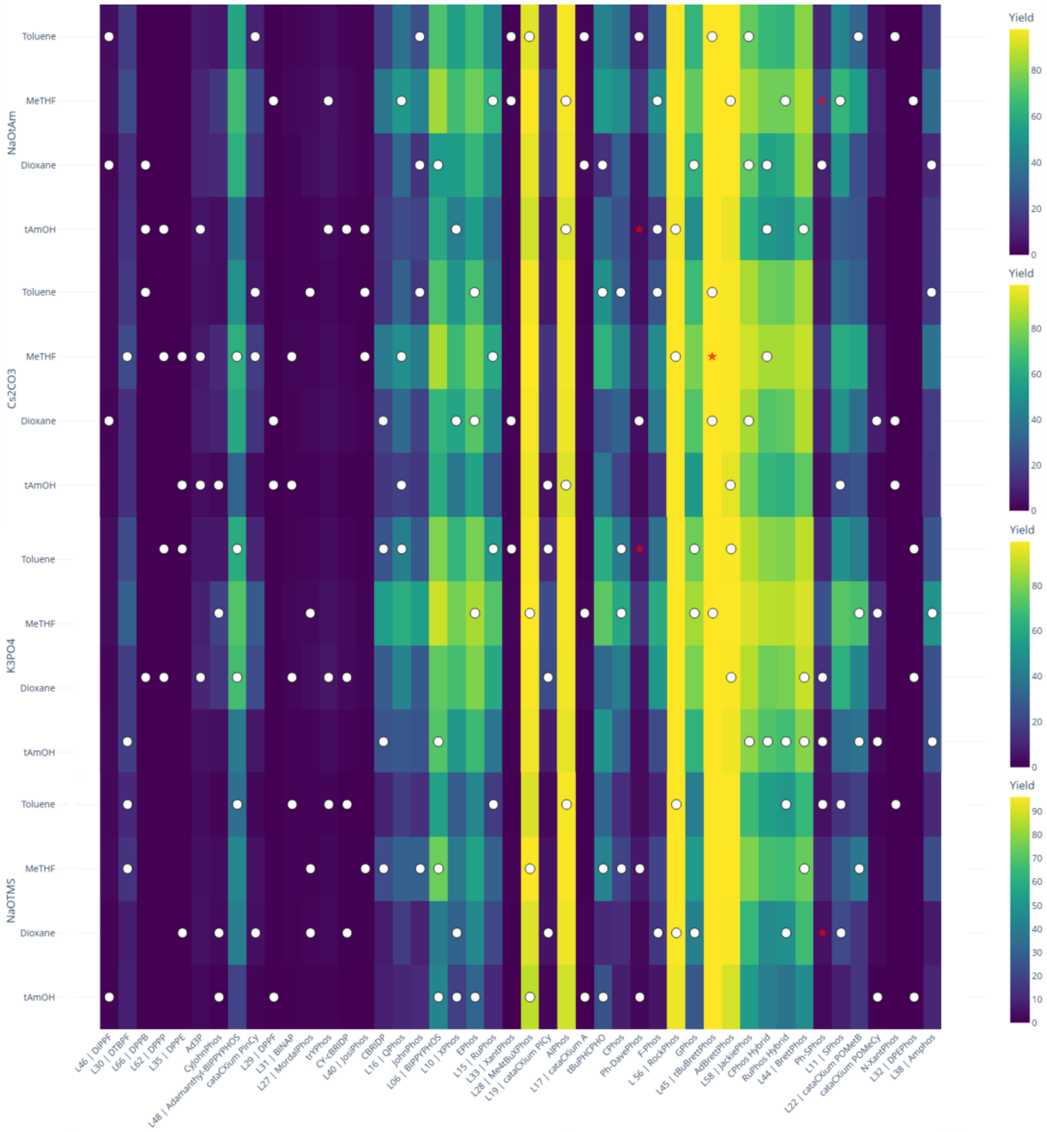

Figure S8: Complementary data visualization after performing the frugal sampling (Sobol, 192 experiments), with bases as the main y axis and solvents as the secondary y axis. White dots = experimental datapoints, red stars = recommended experiments.

ii. Partial dependence plots

The yields (as area percentages) were calculated by taking the integration of the non-limiting reagent into account, which resulted in the product amount being slightly underestimated.

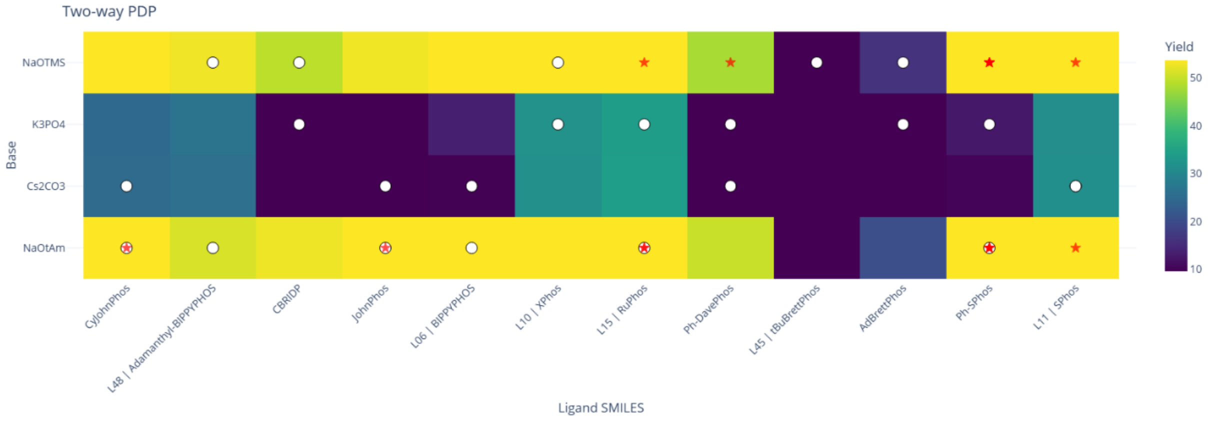

Figure S9: Partial dependence plot between the 12 ligands selected and four bases after the first BO iteration. The heat map values represent the average yield (as area percentages) for all solvent choices. White dots = experimental datapoints, red stars = recommended experiments.

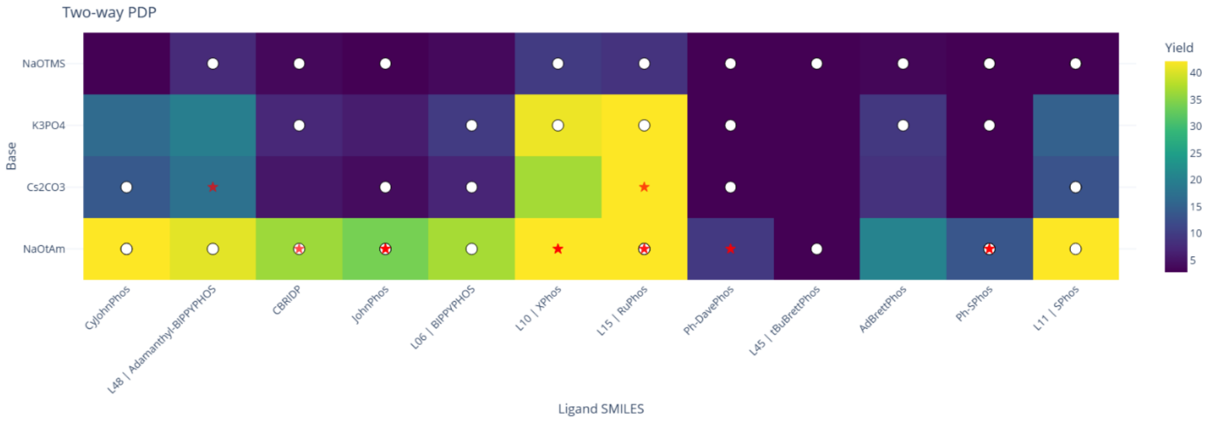

Figure S10: Partial dependence plot between the 12 ligands selected and four bases after the second BO iteration. The heat map values represent the average yield (as area percentages) for all solvent choices. White dots = experimental datapoints, red stars = recommended experiments.

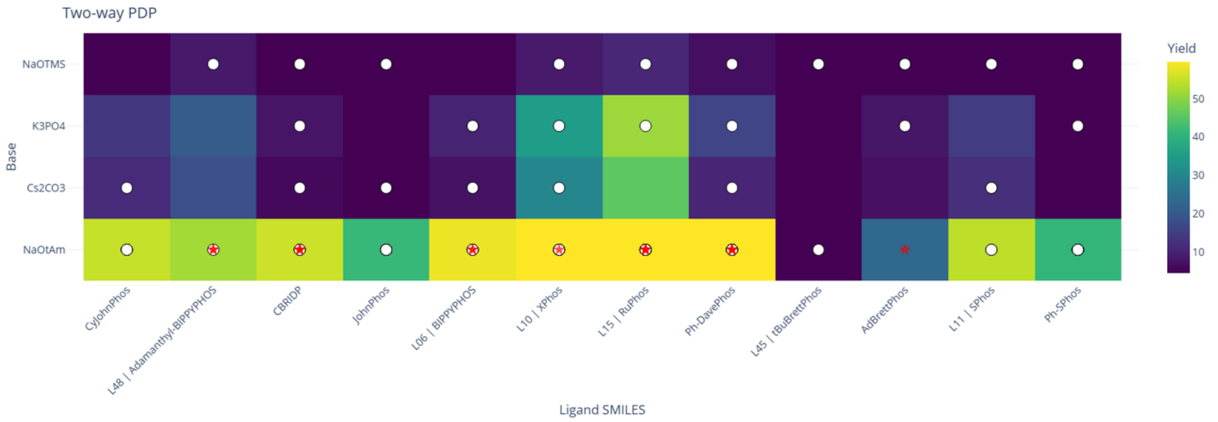

Figure S11: Partial dependence plot between the 12 ligands selected and four bases after the third BO iteration. The heat map values represent the average yield (as area percentages) for all solvent choices. White dots = experimental datapoints, red stars = recommended experiments.

f. DoE for the formation of 13

The effect of some continuous reaction parameters was evaluated in a central composite design (CCD) with the most active precatalyst (**P072_Pd04**), which has an affordable ligand (PhDavePhos), at reduced Pd loading.

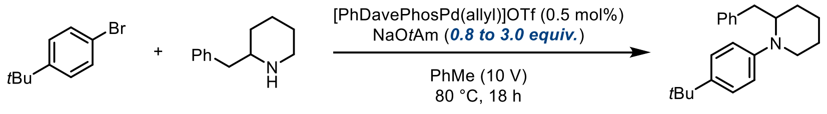

11 **12** *1.1 to 1.6 equiv.* **13**, 99%

In a nitrogen-purged glovebox, NaOtAm (44 – 178 mg, 0.39 – 1.58 mmol, 0.83 – 3.37 equiv.) was added to Xelsius vials followed by the PhDavePhos precatalyst on ChemBeads (234 mg at 10 µmol/g, 0.5 mol%) and the addition of PhMe (1.0 ml). 1-bromo-4-tert-butyl-benzene (81.4 µL, 0.47 mmol, 1.0 equiv.) and 2-benzylpiperidine (85 – 133 µL, 0.48 – 0.74 mmol, 1.02 – 1.58 equiv.) were added sequentially to the appropriate vials. The Xelsius vials were removed from the glovebox and were purged three times using argon/vacuum cycles. The reaction mixtures were stirred at 80 °C, 800 rpm for 18 h. Each reaction mixture was then transferred into a 100-mL volumetric flask and filled with MeCN:H_2O (7:3). The resulting solutions were analyzed by HPLC using Method 1.

Table S17: Factor settings and measured responses as area percent yields for the CCD. [a] The run is a strong outlier and was excluded from the analysis (substoichiometric quantity of NaOtAm was employed).

Std	Run	Space	Factor A NaOtAm (equiv.)	Factor B Amine **12** (equiv.)	Yield of ArBr **11** (%)	Yield of Prod **13** (%)
1	3	Factorial	1,20	1,10	0	99
2	1	Factorial	3,00	1,10	0	99
3	9	Factorial	1,20	1,50	0	98
4	5	Factorial	3,00	1,50	0	98
5[a]	2	Axial	0,83	1,30	15	83
6	6	Axial	3,37	1,30	0	99
7	8	Axial	2,10	1,02	0	99
8	4	Axial	2,10	1,58	0	98
9	7	Center	2,10	1,30	0	98
10	10	Center	2,10	1,30	0	98

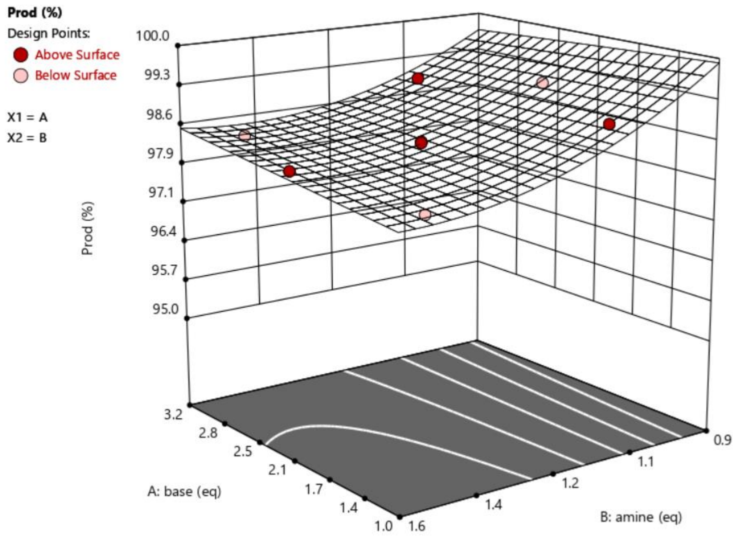

Figure S12: Response surface for the CCD showing stable and quantitative product yield across the design space.

Table S18: ANOVA table for the selected CCD, which indicates a significant quadratic model.

Source	Sum of Squares	df	Mean Square	F-value	p-value	
**Model**	1,2261	4	0,3065	66,60	0,0006	significant
A- NaOtAm	0,0366	1	0,0366	7,96	0,0478	
B-amine	1,0659	1	1,0659	231,60	0,0001	
AB	0,0332	1	0,0332	7,22	0,0548	
B^2	0,1138	1	0,1138	24,73	0,0076	
**Residual**	0,0184	4	0,0046			
Lack of Fit	0,0182	3	0,0061	29,91	0,1334	not significant
Pure Error	0,0002	1	0,0002			
**Cor Total**	1,2445	8				

Table S19: Fit statistics, which confirm that a predictive model can be used to navigate the design space.

**Std. Dev.**	0,0678	**R^2**	0,9852
**Mean**	98,60	**Adjusted R^2**	0,9704
**C.V. %**	0,0688	**Predicted R^2**	0,8820
		**Adeq Precision**	23,9562

g. Isolation and characterization of 13

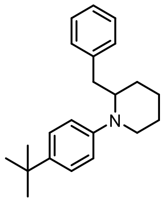

Isolation of an analytical sample was performed prior to optimization activities.

In a Xelsius vial was charged 1-bromo-4-tert-butyl-benzene (100 mg, 0.5 mmol, 1.0 equiv.) followed by *t*AmOH (1 mL), NaOtAm (40% in toluene, 0.3 mL, 0.9 mmol, 2.0 equiv.) and 2-benzylpiperidine (109 mg, 0.6 mmol, 1.3 equiv.). The mixture was degassed with 3 cycles of vacuum/argon and [Pd(crotyl)(QPhos)]Cl (10 mg, 11 µmol, 2.5 mol%) was added. The vial was then stirred at 80 °C for 18 h. After allowing the reaction mixture to return to ambient temperature, it was diluted with DMF/H_2O and purified by reverse phase HPLC under acidic conditions to afford an analytical sample of the formate salt of **13**.

^{1}H NMR (400 MHz, D_6-DMSO) δ ppm 7.71 – 7.63 (b, 2H), 7.59 - 7.57 (m, 2H), 7.30 - 7.23 (m, 2H), 7.17 - 7.23 (m, 1H), 7.06 - 7.04 (m, 2H), 4.16 - 4.05 (m, 1H), 3.66 - 3.47 (m, 2H), 2.68 - 2.55 (m, 2H),1.97 - 1.70 (m, 5H), 1.68 - 1.54 (m, 1H), 1.30 (s, 9H).

^{13}C NMR (100 MHz, D_6-DMSO): 158.6, 139.4, 136.5, 129.6, 128.8 (2C), 128.5 (2C), 126.9 (2C), 126.6 (2C), 120.8, 117.9, 115.0, 112.0, 64.8, 36.5, 34.4, 30.9, 27.9, 23.5, 20.8.

HRMS (ESI$^+$) m/z: $[M + H]^+$ Calcd for $C_{22}H_{30}N$ 308.2373; Found 308.2372

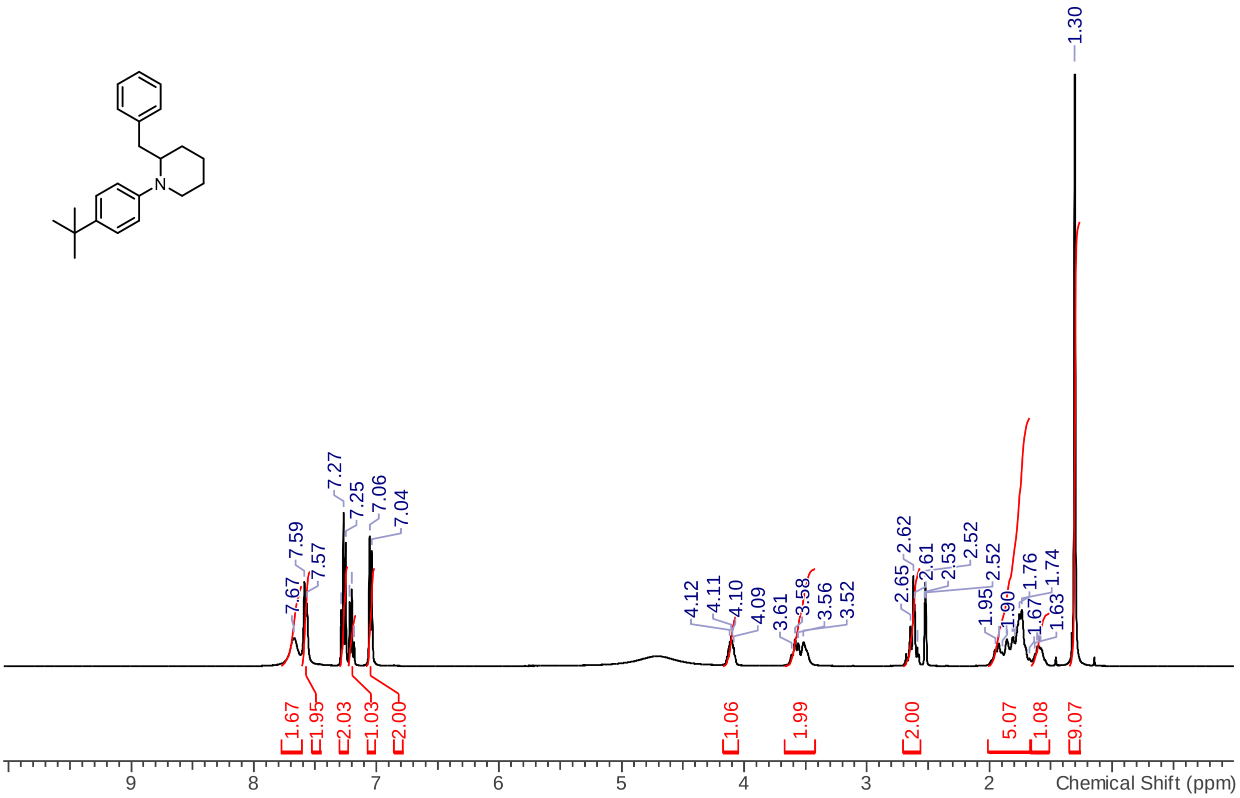

*Figure S13: ^{1}H NMR (400 MHz, D_6-DMSO) of **13***

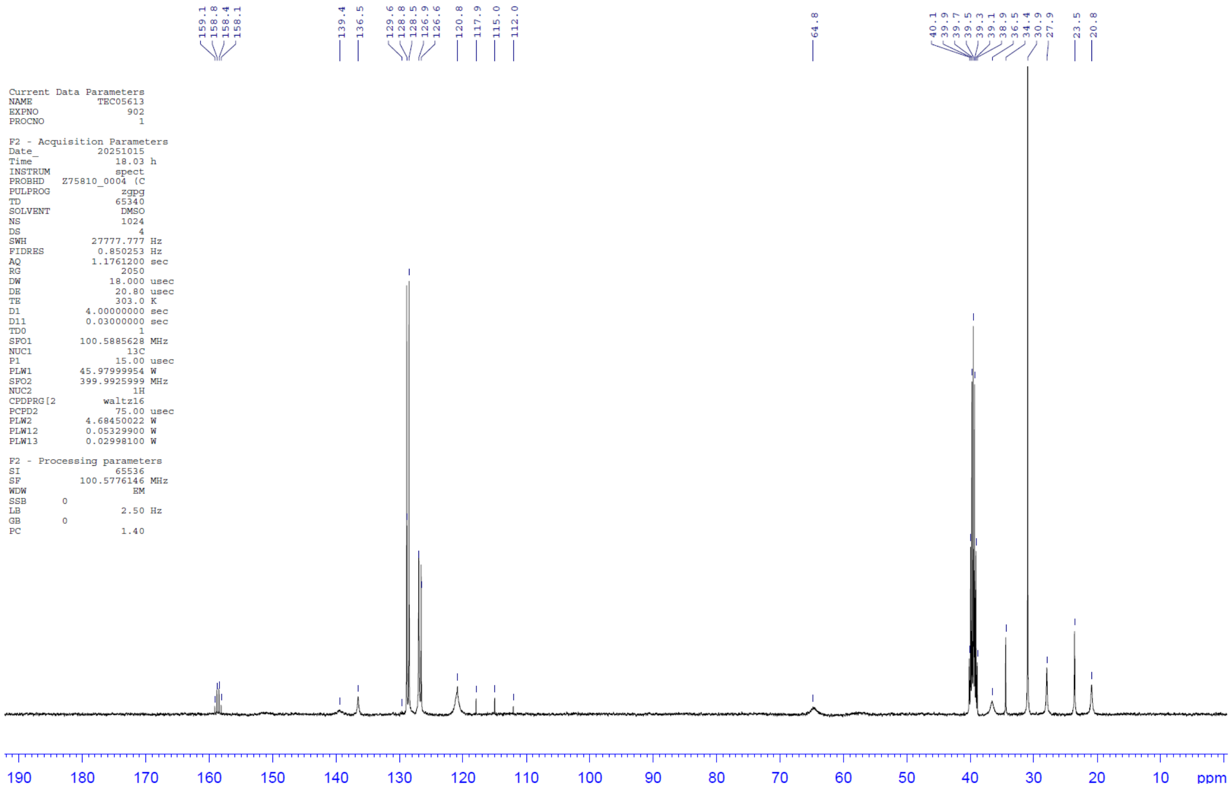

*Figure S14: ^{13}C NMR (100 MHz, D_6-DMSO) of **13**.*
